# Ufd1-Npl4 Recruit Cdc48 for Disassembly of Ubiquitylated CMG Helicase at the End of Chromosome Replication

**DOI:** 10.1016/j.celrep.2025.116569

**Published:** 2025-11-06

**Authors:** Marija Maric, Progya Mukherjee, Michael H. Tatham, Ronald Hay, Karim Labib

## Main text

(Cell Reports *18*, 3033–3042; March 28, 2017)

The authors have identified an assembly error in Figure 2D of their paper published in 2017. The immunoblotting panel for Mcm3 extracts of cells at 37°C was inadvertently duplicated for that of Cdc48 extracts of cells at 37°C. This error has no impact on the conclusions of the article and has been examined by the Research Integrity Group of the School of Life Sciences at the University of Dundee. The corrected panel is below, and source data have been published at Mendeley Data (doi: https://doi.org/10.17632/k2x87hn47g.1). The authors apologize for the error.

**Figure 2D:**
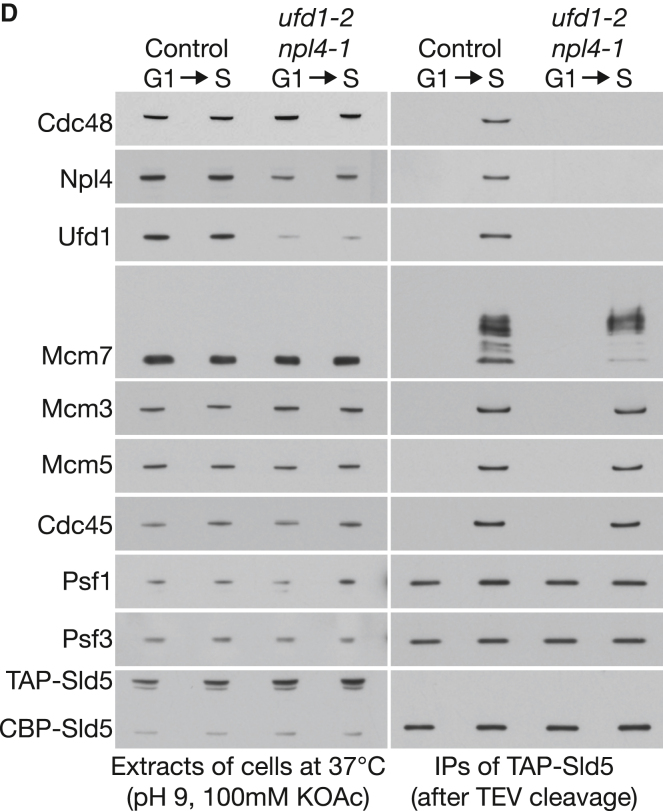
Ufd1-Npl4 Are Required to Recruit Cdc48 to the CMG Helicase

