# Supplementary material for: Ufd1-Npl4 Recruit Cdc48 for Disassembly of Ubiquitylated CMG Helicase at the End of Chromosome Replication
Source: Cell Rep. 2017 Mar 28;18(13):3033–42. doi: 10.1016/j.celrep.2017.03.020 (PMC5382235; doi:10.1016/j.celrep.2017.03.020)
Supplement: Document S1. Figures S1–S4 and Tables S1 and S2 [file mmc1.pdf]

Cell Reports, Volume *18*

Supplemental Information

Ufd1-Npl4 Recruit Cdc48 for Disassembly of Ubiquitylated CMG Helicase at the End of Chromosome Replication

Marija Maric, Progya Mukherjee, Michael H. Tatham, Ronald Hay, and Karim Labib

Maric et al Supplementary Figure 1, related to Figure 1

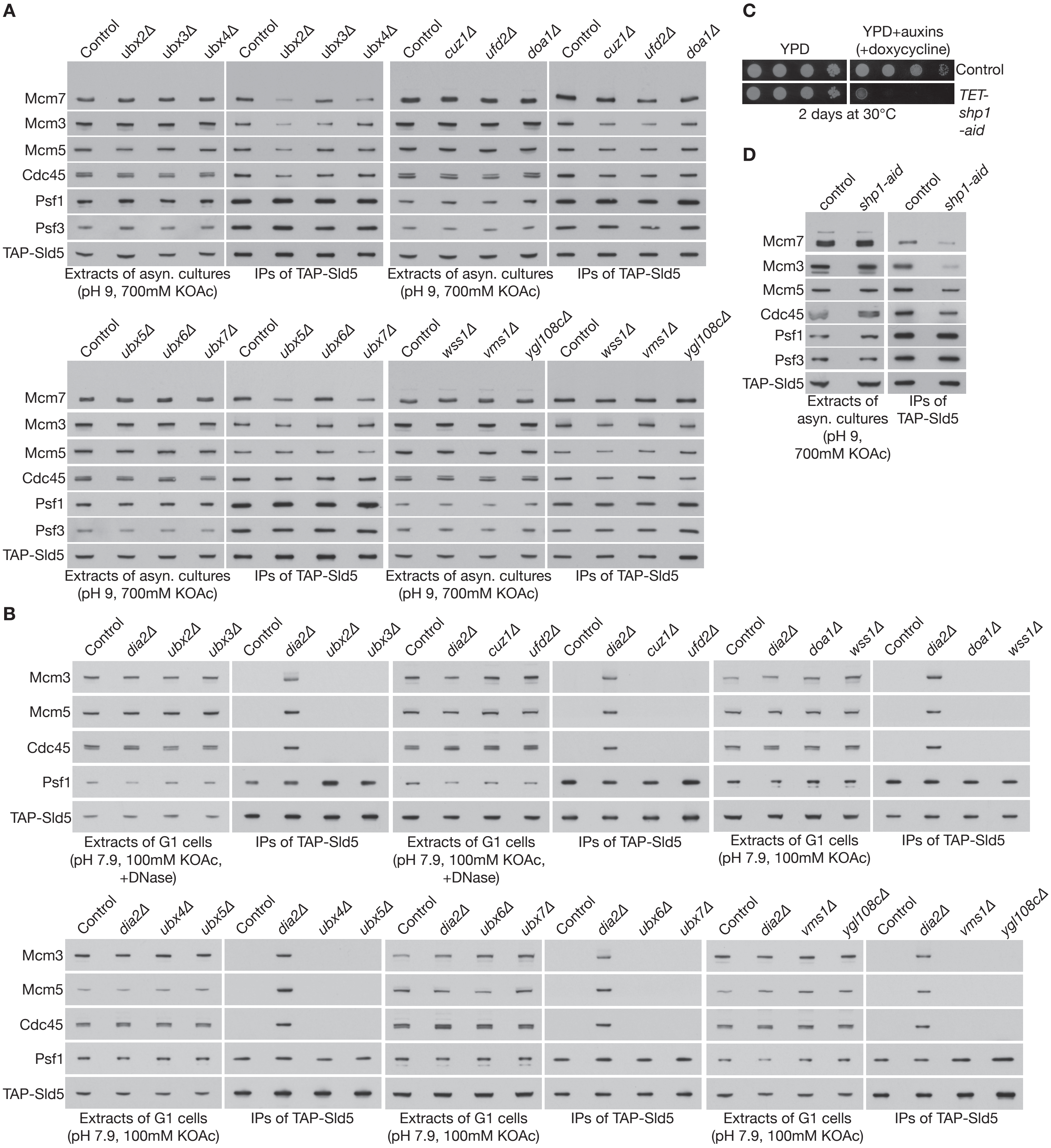

Screen for adaptors of Cdc48 that are required for disassembly of ubiquitylated CMG helicase at the end of chromosome replication. (**A**) Asynchronous cultures of the indicated strains were grown at 30°C. The presence of ubiquitylated CMG helicase was monitored by immunoprecipitation of TAP-Sld5 from high salt extracts, as in Figure 1 for *ufd1-2* and *npl4-1* (which provide a positive control for the factors shown here). (**B**) The indicated strains were grown at 30°C and then arrested in G1-phase. The persistence of old CMG complexes was monitored by immunoprecipitation of TAP-Sld5 from cell extracts - *dia2Δ* provided a positive control for defective CMG disassembly. (**C**) Comparison of growth of control cells (YMM573) and TET-shp1-aid (YMM591) on rich medium (YPD) in the presence or absence of auxins (to induce degradation of Shp1-aid) and doxycycline (to repress expression of *shp1-aid* from the *TET* promoter). (**D**) Cells were grown at 30°C before addition of doxycycline and auxin for 3 hours (control strain YSS47 and *TET-shp1-aid* strain YMM591). The presence of ubiquitylated CMG helicase was assayed as above.

Maric et al Supplementary Figure 2, related to Figure 1

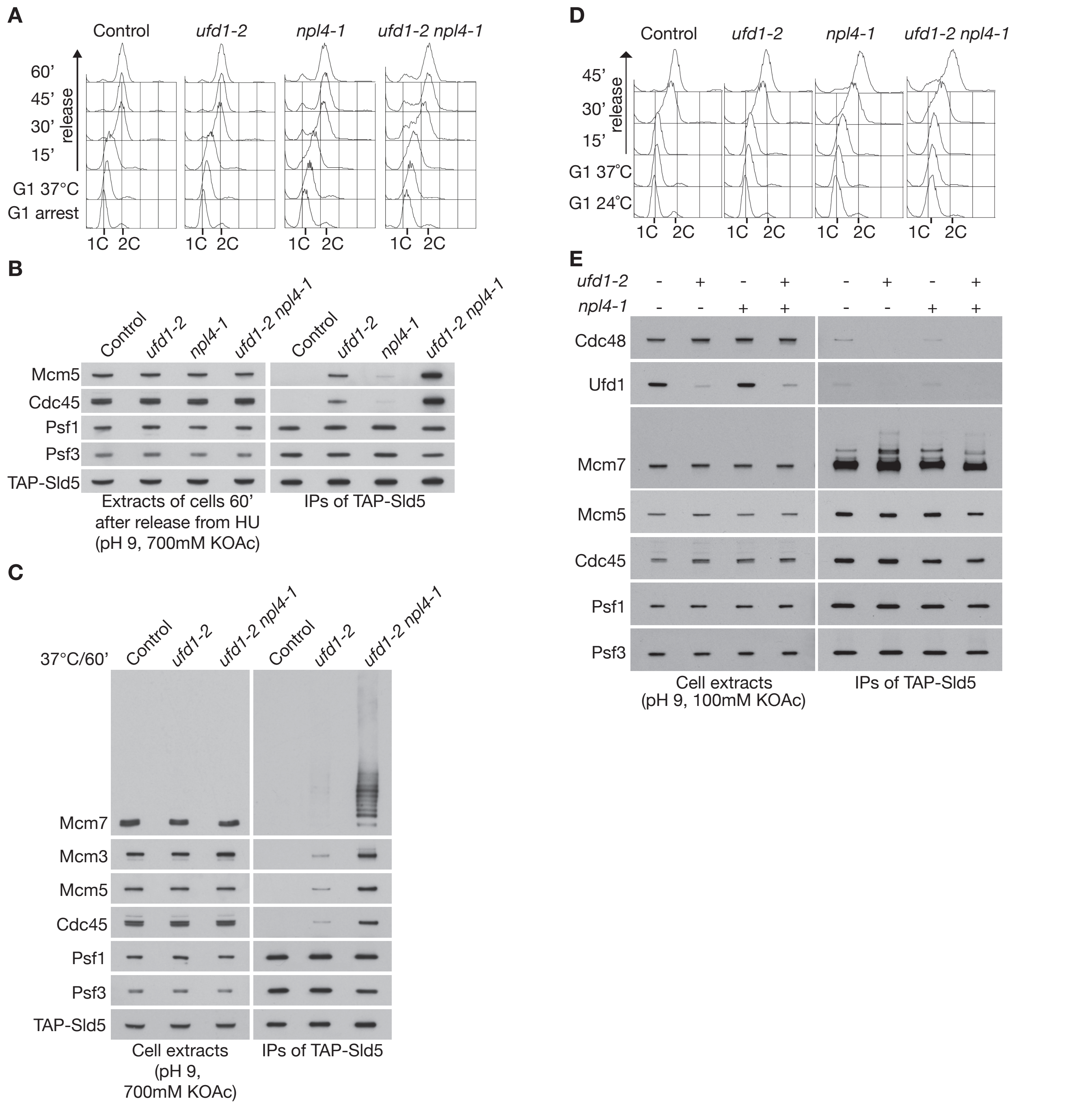

Inactivation of both Ufd1 and Npl4 is necessary to block CMG disassembly efficiently at the end of chromosome replication, though the *ufd1-2* mutation leads to displacement of Cdc48 from the ubiquitylated CMG helicase.
(**A**) Control cells (YSS47), *ufd1-2* (YMM306), *npl4-1* (YMM308) and *ufd1-2 npl4-1* (YMM574) were synchronized in G1-phase with mating pheromone at 24°C and then shifted to 37°C for 60’, before release into fresh medium lacking mating pheromone. (**B**) Samples were taken at the 60’ timepoint, after chromosome replication had been completed and CMG was monitored as above, by immunoprecipitation of the Sld5 subunit of GINS from high salt extracts. The *ufd1-2 npl4-1* double mutant shows an enhanced defect in CMG disassembly at the end of S-phase, compared to the *ufd1-2* or *npl4-1* single mutants. (**C**) Re-run of selected samples from (b) to illustrate the accumulation of CMG with ubiquitylated Mcm7 subunit in the *ufd1-2 npl4-1* double mutant. (**D**) Control (YSS47), *ufd1-1* (YMM306), *npl4-1* (YMM308) and *ufd1-2 npl4-1* cells (YMM574) were synchronized in G1-phase at 24°C and then shifted to 37°C for 60’ before release into S-phase for 15’, as described above for Figure 2c-d. (**E**) The association of Ufd1 and Cdc48 with CMG was monitored as above.

Maric et al Supplementary Figure 3, related to Figure 3

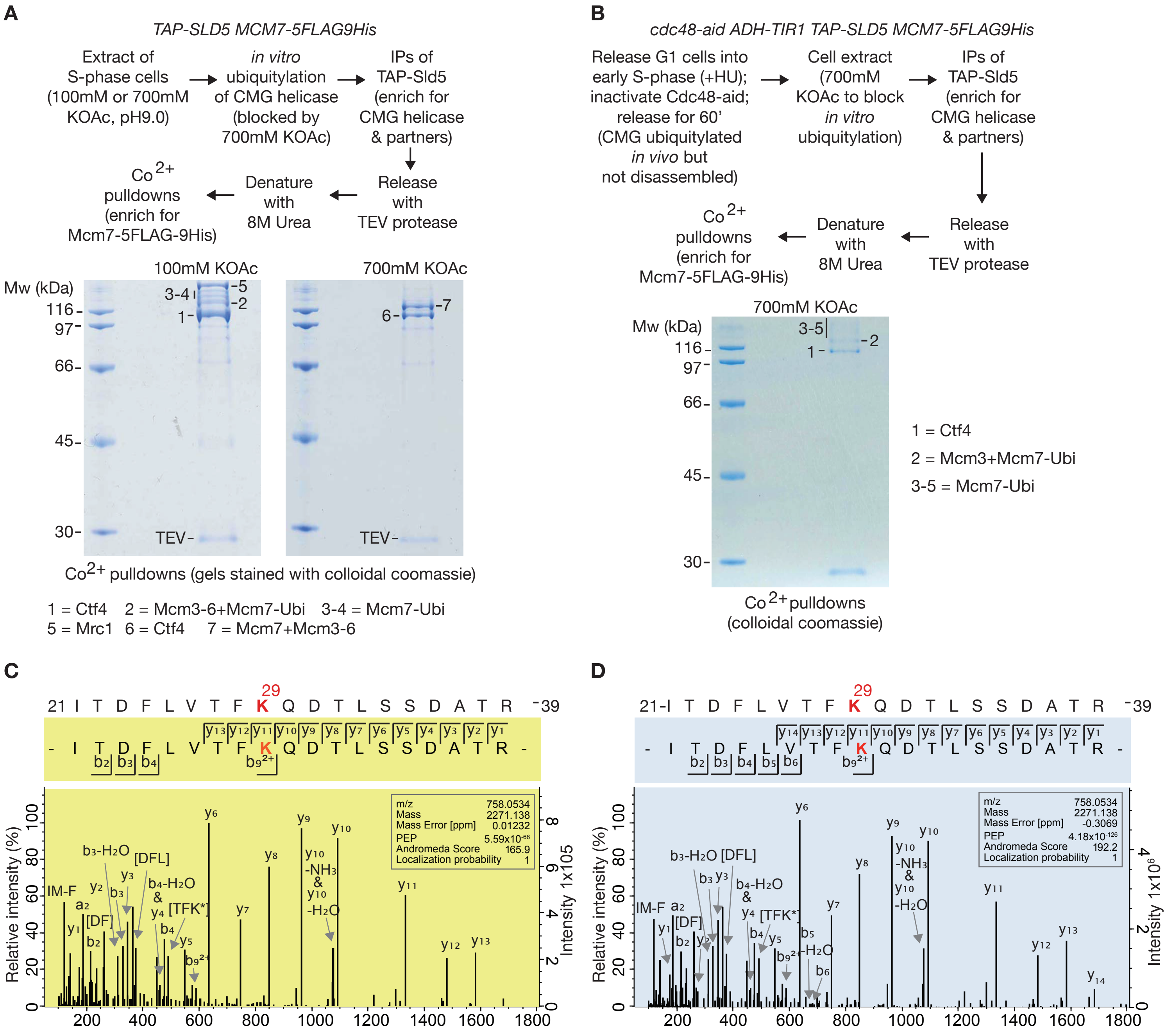

Lysine 29 of Mcm7 is the major site of CMG ubiquitylation both *in vitro* and *in vivo*.

(**A**) Scheme to enrich for ubiquitylated Mcm7 from isolated CMG helicase complexes. After isolation of GINS (and thus CMG) by immunoprecipitation of TAP-Sld5 from extracts of S-phase cells (YGDP483), we released the bound complexes with TEV protease, denatured the material with 8M urea, and then isolated Mcm7-5FLAG9His on cobalt-coated magnetic beads. We compared low salt extracts that supported *in vitro* ubiquitylation with high salt extracts in which ubiquitylation was blocked. Note that several components of CMG, or associated factors, bound non-specifically in their denatured form to the cobalt-coated beads (Ctf4, Mrc1, Mcm3, Mcm6 - the presence of the latter factors was greatly reduced in high salt extracts). The purified material was then resolved in a 10% gel, and the indicated bands excised for mass spectrometry analysis. (**B**) Similar analysis of Mcm7 from in vivo ubiquitylated CMG helicase. A culture of *cdc48-aid* cells (YMM228) was synchronised in G1-phase with mating pheromone and then arrested in early S-phase with 0.2M hydroxyurea, before depletion of Cdc48-aid by addition of auxin for 60'. Cells were then washed into fresh medium lacking hydroxyurea, and incubated for a further 60'. (**C**) Following in-gel digestion of in vitro ubiquitylated Mcm7 with trypsin and GluC, mass spectrometry analysis indicated that K29 was the only detectable site of Gly-Gly modification, indicative of ubiquitylation. The panel shows the annotated MS/MS spectrum of the GluC/Trypsin peptide that is diagnostic of ubiquitylated Mcm7-K29 (only the peaks with >10% of maximum intensity are annotated; annotations in square brackets show internal peptide fragments). (**D**) *In vivo* ubiquitylated Mcm7 was processed in the same way, and once again K29 was the only detectable site of ubiquitylation.

Maric et al Supplementary Figure 4, related to Figure 4

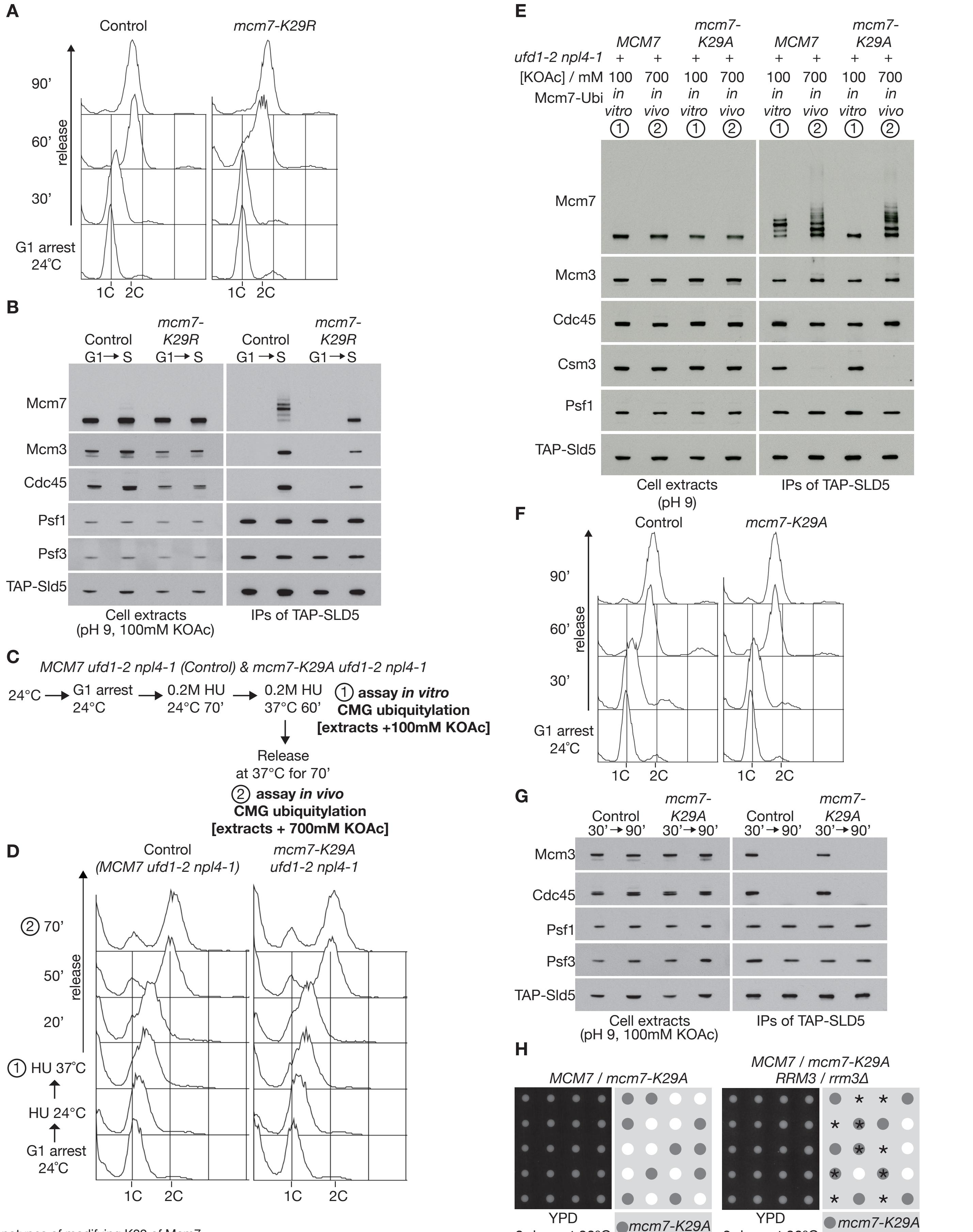

Phenotypes of modifying K29 of Mcm7.

(**A**) Control (YGDP483) and *mcm7-K29R* (YPM160) yeast cells were synchronised in G1-phase at 24°C andthen released into S-phase for 20’. (**B**) *In vitro* ubiquitylation of the CMG helicase in extracts of S-phase cells was monitored as above.

(**C**) *MCM7 ufd1-2 npl4-1* control cells (YPM81) and *mcm7-K29a ufd1-2 npl4-1* (YPM94) were grown as in Figure 1c. Cells were synchronized in G1-phase at 24°C and then released into S-phase in the presence of 0.2M hydroxyurea for 70’, to allow for CMG assembly at forks from early origins of replication. The cells were then shifted to 37°C for 60’ to inactivate Ufd1-Npl4, before washing into fresh medium lacking hydroxyurea, to allow cells to complete chromosome replication. Sample 1 corresponded to HU arrested cells at 37°C that lacked in vivo CMG ubiquitylation (defective progression of DNA replication forks blocked the termination of DNA replication), and was used to prepare ‘low-salt extracts’ in order to monitor in vitro ubiquitylation of CMG helicase. In contrast, Sample 2 was taken at the end of the experiment and corresponded to cells that had completed chromosome replication in the absence of CMG disassembly (due to inactivation of Ufd1-Npl4); this sample was used to prepare ‘high-salt’ extracts in order to monitor in vivo ubiquitylated CMG helicase. (**D**) DNA content was monitored throughout the experiment by flow cytometry. (**E**) The CMG helicase was isolated from cell extracts by immunoprecipitation of the Sld5 subunit of GINS, revealing that the mcm7-K29A mutation abolished in vitro CMG ubiquitylation in cell extracts but did not prevent in vivo ubiquitylation at the end of chromosome replication. (**F**) Synchronised cultures of control (YPM81) and *mcm7-K29A* cells (YPM94) were generated as in Supplementary Fig. 6. (**G**) CMG disassembly was monitored by immunoprecipitation of the Sld5 subunit of GINS in mid-S-phase (30’ release from G1-phase) and following the completion of chromosome replication (90’). (**H**) Tetrad analysis of the indicated diploid strains (YMM476 and YMM482) shows that *mcm7-K29A* grows like control cells and is not synthetic lethal with *rrm3Δ*.

Maric et al Supplementary Table 1, related to Figure 3

	In vitro Mcm7-Ubi		*In vivo* Mcm7-Ubi	
**Fragment**	**Mass Deviation (ppm)**	**Mass (Da)**	**Mass Deviation (ppm)**	**Mass (Da)**
y_1	1.6420	175.1187	3.9000	175.1183
a_2	1.6948	187.1438	3.8979	187.1434
b_2-H_2O	1.1290	197.1282	3.5890	197.1277
b_2	1.7325	215.1386	3.8394	215.1382
y_2-NH_3			4.2236	259.1390
y_2	0.8279	276.1664	1.6296	276.1662
b_3-H_2O	3.0079	312.1545	5.2404	312.1538
b_3	4.6527	330.1644	7.3189	330.1635
y_3	1.8004	347.2031	3.8534	347.2024
b_4-H_2O	1.9465	459.2229		
y_4	1.9004	462.2298	3.9021	462.2289
b_4	1.5331	477.2336	4.0143	477.2325
y_5	0.6584	549.2624	3.6980	549.2607
b_5-H_2O			0.8942	572.3074
$b_9^{(2+)}$	0.1526	590.3240	2.8573	590.3224
y_6	1.8132	636.2936	3.9031	636.2923
b_6			1.6684	689.3857
y_7	2.8235	749.3767	4.2774	749.3756
y_8	2.2385	850.4246	3.6823	850.4234
y_9	2.2967	965.4512	4.0344	965.4495
y_{10}-H_2O	1.9836	1075.4993	3.6085	1075.4976
y_{10}-NH_3	-1.4574	1076.4870	0.7890	1076.4846
y_{10}	1.8315	1093.5100	3.7835	1093.5079
y_{11}	1.3399	1335.6481	3.1013	1335.6458
y_{12}-H_2O			0.9205	1464.7064
y_{12}-NH_3			2.7067	1465.6878
y_{12}	1.4668	1482.7161	3.3160	1482.7134
y_{13}-H_2O			3.1593	1565.7505
y_{13}-NH_3			-0.5000	1566.7402
y_{13}	0.6992	1583.7649	3.2874	1583.7608
y_{14}			1.1983	1682.8324

Summary of MS data for mapping of Mcm7 ubiquitylation sites.
The table shows the annotated MS/MS data for the two spectra in Figure S3C-D.

Strain	Genotype
W303-1	*MAT***a** *ade2-1 ura3-1 his3-11,15 trp1-1 leu2-3,112 can1-100* / *MAT*α *ade2-1 ura3-1 his3-11,15 trp1-1 leu2-3,112 can1-100*
YASD375	*MAT***a** *TAP-SLD5 (kanMX) pep4Δ::URA3 ADE2*
YGDP483	*MAT***a** *TAP-SLD5 (kanMX) MCM7-5FLAG9His (hphNT) pep4Δ::URA3 ADE2*
YMM120	*MAT***a** *leu2::pRS305-GAL-MCM7 mcm7-td (K.l.TRP1) GAL-UBR1 (URA3) TAP-SLD5 (kanMX) pep4Δ::ADE2*
YMM228	*MAT***a** *cdc48-aid (hphNT) ADH1-OsTIR1 (URA3 & K.l.TRP1) TAP-SLD5 (kanMX) pep4Δ::URA3 ADE2*
YMM233	*MAT***a** *leu2::pRS305-GAL-mcm7-K624A,K637A,K648A,K672A,K678A mcm7-td (K.l.TRP1) GAL-UBR1 (URA3) TAP-SLD5 (kanMX) pep4Δ::ADE2*
YMM238	*MAT***a** *leu2::pRS305-GAL-mcm7-K442A,K449R,K471A,K475R,K486R,K499R,K528A,K550A mcm7-td (K.l.TRP1) GAL-UBR1 (URA3) TAP-SLD5 (kanMX) pep4Δ::ADE2*
YMM240	*MAT***a** *leu2::pRS305-GAL-mcm7-K733A,K735A,K743A,K749A,K750A,K757A,K767A,K822A mcm7-td (K.l.TRP1) GAL-UBR1 (URA3) TAP-SLD5 (kanMX) pep4Δ::ADE2*
YMM242	*MAT***a** *leu2::pRS305-GAL-mcm7-K29A,K59A,K62A,K69A,K92A,K130A,K135A mcm7-td (K.l.TRP1) GAL-UBR1 (URA3) TAP-SLD5 (kanMX) pep4Δ::ADE2*
YMM244	*MAT***a** *leu2::pRS305-GAL-mcm7-K217A,K218A,K223A,K231A,K252R,K295A,K314R,K364A, K367A,K385A,K386R,K387A mcm7-td (K.l.TRP1) GAL-UBR1 (URA3) TAP-SLD5 (kanMX) pep4Δ::ADE2*
YMM306	*MAT***a** *ufd1-2 TAP-SLD5 (kanMX) pep4Δ::URA3 ADE2*
YMM308	*MAT***a** *npl4-1 TAP-SLD5 (kanMX) pep4Δ::URA3 ADE2*
YMM377	*MAT***a** *leu2::pRS305-GAL-mcm7-K29A,K59A,K62A mcm7-td (K.l.TRP1) GAL-UBR1 (URA3) TAP-SLD5 (kanMX) pep4Δ::ADE2*
YMM379	*MAT***a** *leu2::pRS305-GAL-mcm7-K29A,K59A,K62A,K69A mcm7-td (K.l.TRP1) GAL-UBR1 (URA3) TAP-SLD5 (kanMX) pep4Δ::ADE2*
YMM396	*MAT***a** *leu2::pRS305-GAL-mcm7-K29A,K59A,K62A,K69A,K92A mcm7-td (K.l.TRP1) GAL-UBR1 (URA3) TAP-SLD5 (kanMX) pep4Δ::ADE2*
YMM397	*MAT***a** *leu2::pRS305-GAL-mcm7-K130A,K135A mcm7-td (K.l.TRP1) GAL-UBR1 (URA3) TAP-SLD5 (kanMX) pep4Δ::ADE2*
YMM408	*MAT***a** *ubx5Δ::K.l.TRP1 TAP-SLD5 (kanMX) pep4Δ::ADE2*
YMM412	*MAT***a** *ufd2Δ::K.l.TRP1 TAP-SLD5 (kanMX) pep4Δ::ADE2*
YMM435	*MAT***a** *leu2::pRS305-GAL-mcm7-K29A mcm7-td (K.l.TRP1) GAL-UBR1 (URA3) TAP-SLD5 (kanMX) pep4Δ::ADE2*
YMM437	*MAT***a** *leu2::pRS305-GAL-mcm7-K59A mcm7-td (K.l.TRP1) GAL-UBR1 (URA3) TAP-SLD5 (kanMX) pep4Δ::ADE2*
YMM439	*MAT***a** *leu2::pRS305-GAL-mcm7-K62A mcm7-td (K.l.TRP1) GAL-*

	UBR1 (URA3) TAP-SLD5 (kanMX) pep4Δ::ADE2
YMM458	*MAT***a** *cuz1Δ::K.l.TRP1 TAP-SLD5 (kanMX) pep4Δ::ADE2*
YMM460	*MAT***a** *ubx4Δ::K.l.TRP1 TAP-SLD5 (kanMX) pep4Δ::ADE2*
YMM476	*MAT***a** / *MATα MCM7 / mcm7-K29A-5FLAG9His (hphNT)*
YMM482	*MAT***a** / *MATα MCM7 / mcm7-K29A-5FLAG9His (hphNT) RRM3 / rrm3Δ*
YMM493	*MAT***a** *mcm7-K29A-5FLAG9His (hphNT) TAP-SLD5 (kanMX) pep4Δ::ADE2*
YMM495	*MAT***a** *vms1Δ::K.l.TRP1 TAP-SLD5 (kanMX) pep4Δ::ADE2*
YMM503	*MAT***a** *ygl108cΔ::K.l.TRP1 TAP-SLD5 (kanMX) pep4Δ::ADE2*
YMM510	*MAT***a** / *MATα UBX5 / ubx5Δ::K.l.TRP1 DIA2 / dia2Δ::HIS3MX*
YMM511	*MAT***a** / *MATα UFD2 / ufd2Δ::K.l.TRP1 DIA2 / dia2Δ::HIS3MX*
YMM512	*MAT***a** / *MATα CUZ1 / cuz1Δ::K.l.TRP1 DIA2 / dia2Δ::HIS3MX*
YMM513	*MAT***a** / *MATα YGL108C / ygl108cΔ::K.l.TRP1 DIA2 / dia2Δ::HIS3MX*
YMM530	*MAT***a** / *MATα UBX2 / ubx2Δ::hphNT DIA2 / dia2Δ::HIS3MX*
YMM531	*MAT***a** / *MATα UBX3 / ubx3Δ::HIS3MX DIA2 / dia2Δ::HIS3MX*
YMM532	*MAT***a** / *MATα UBX6 / ubx6Δ::URA3CP DIA2 / dia2Δ::HIS3MX*
YMM533	*MAT***a** / *MATα UBX7 / ubx7Δ::HIS3MX DIA2 / dia2Δ::HIS3MX*
YMM534	*MAT***a** / *MATα UBX4 / ubx4Δ::K.l.TRP1 DIA2 / dia2Δ::HIS3MX*
YMM543	*MAT***a** *ubx2Δ::hphNT TAP-SLD5 (kanMX) pep4Δ::ADE2*
YMM545	*MAT***a** *ubx3Δ::HIS3MX TAP-SLD5 (kanMX) pep4Δ::ADE2*
YMM547	*MAT***a** *ubx6Δ::URA3CP TAP-SLD5 (kanMX) pep4Δ::ADE2*
YMM549	*MAT***a** *ubx7Δ::HIS3MX TAP-SLD5 (kanMX) pep4Δ::ADE2*
YMM555	*MAT***a** / *MATα DOA1 / doa1Δ::hphNT DIA2 / dia2Δ::HIS3MX*
YMM556	*MAT***a** / *MATα WSS1 / wss1Δ::hphNT DIA2 / dia2Δ::HIS3MX*
YMM557	*MAT***a** *doa1Δ::hphNT TAP-SLD5 (kanMX) pep4Δ::ADE2*
YMM559	*MAT***a** *wss1Δ::hphNT TAP-SLD5 (kanMX) pep4Δ::ADE2*
YMM561	*MAT***a** / *MATα VMS1 / vms1Δ::K.l.TRP1 DIA2 / dia2Δ::HIS3MX*
YMM562	*MAT***a** / *MATα UFD1 / ufd1-2 DIA2 / dia2Δ::HIS3MX*
YMM563	*MAT***a** / *MATα NPL4 / npl4-1 DIA2 / dia2Δ::HIS3MX*
YMM573	*MATα leu2::pCM244 ADH1-OsTIR1 (URA3 & K.l.TRP1) TAP-SLD5 pep4Δ::ADE2*
YMM574	*MAT***a** *ufd1-2 npl4-1 MCM7-5FLAG9His (hphNT) TAP-SLD5 (kanMX) pep4Δ::URA3 ADE2*
YMM591	*MAT***a** *TET-shp1-aid (kanMX & hphNT) leu2::pCM244 ADH1-OsTIR1 (URA3 & K.l.TRP1) TAP-SLD5 (kanMX) pep4Δ::ADE2*
YPM81	*MAT***a** *ufd1-2 npl4-1 TAP-SLD5 (kanMX) pep4Δ::ADE2*
YPM94	*MAT***a** *ufd1-2 npl4-1 mcm7-K29A-5FLAG9His (hphNT) TAP-SLD5 (kanMX) pep4Δ::ADE2*
YPM160	*MAT***a** *mcm7-K29R-5FLAG9His (hphNT) TAP-SLD5 (kanMX) pep4Δ::ADE2*
YSS47	*MAT***a** *TAP-SLD5 (kanMX) pep4Δ::ADE2*
YSS125	*MAT***a** *mcm7-td (K.l.TRP1) GAL-UBR1 (URA3) TAP-SLD5 (kanMX) pep4Δ::ADE2*
YTM572	*MAT***a** *dia2Δ::HIS3MX TAP-SLD5 (kanMX) pep4Δ::ADE2*

Supplementary Table 2, related to Figures 1-4

Yeast strains used in this study. All strains are based on the W303 background.
