# Supplementary material for: Ufd1-Npl4 Recruit Cdc48 for Disassembly of Ubiquitylated CMG Helicase at the End of Chromosome Replication
Source: Cell Rep. 2017 Mar 28;18(13):3033–42. doi: 10.1016/j.celrep.2017.03.020 (PMC5382235; doi:10.1016/j.celrep.2017.03.020)
Supplement: Document S2. Article plus Supplemental Information [file mmc2.pdf]

Report

Cell Reports

# Ufd1-Npl4 Recruit Cdc48 for Disassembly of Ubiquitylated CMG Helicase at the End of Chromosome Replication

## Graphical Abstract

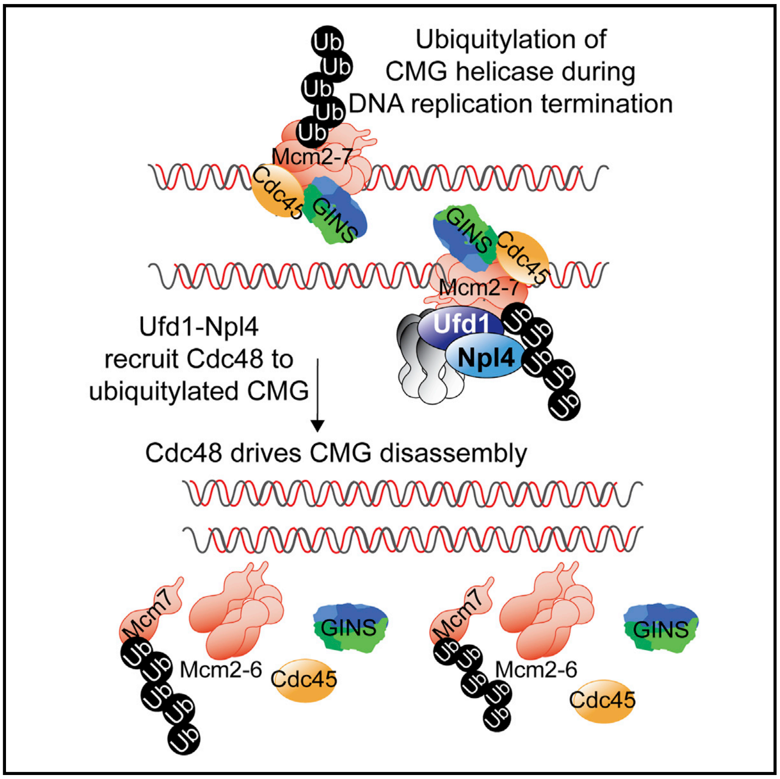


## Highlights

- Ufd1-Npl4 are required for CMG helicase disassembly during DNA replication termination
- Ufd1-Npl4 recruit Cdc48 to ubiquitylated CMG helicase
- Budding yeast CMG helicase is ubiquitylated on K29 of its Mcm7 subunit
- Ubiquitylation of Mcm7-K29 is required in vitro for recruitment of Ufd1-Npl4-Cdc48

## Authors

Marija Maric, Progya Mukherjee, Michael H. Tatham, Ronald Hay, Karim Labib

## Correspondence

kpmlabib@dundee.ac.uk

## In Brief

Disassembly of the CMG helicase is the key regulated step during DNA replication termination in eukaryotes, driven by CMG ubiquitylation and the Cdc48 segregase. Maric et al. find that the Ufd1-Npl4 heterodimer is essential for CMG disassembly in budding yeast and is required to recruit Cdc48 to the ubiquitylated helicase.

Maric et al., 2017, Cell Reports *18*, 3033–3042
March 28, 2017 © 2017 The Author(s).
http://dx.doi.org/10.1016/j.celrep.2017.03.020

OPEN ACCESS
CellPress

# Ufd1-Npl4 Recruit Cdc48 for Disassembly of Ubiquitylated CMG Helicase at the End of Chromosome Replication

Marija Maric,[1,3] Progya Mukherjee,[1] Michael H. Tatham,[2] Ronald Hay,[2] and Karim Labib[1,4,*]
[1]MRC Protein Phosphorylation and Ubiquitylation Unit, Sir James Black Centre, School of Life Sciences, University of Dundee, Dow Street, Dundee DD1 5EH, UK
[2]Gene Regulation and Expression Division, School of Life Sciences, University of Dundee, Dow Street, Dundee DD1 5EH, UK
[3]Present address: The Francis Crick Institute, 1 Midland Road, London NW1 1AT, UK
[4]Lead Contact
*Correspondence: kpmlabib@dundee.ac.uk
http://dx.doi.org/10.1016/j.celrep.2017.03.020

## SUMMARY

**Disassembly of the Cdc45-MCM-GINS (CMG) DNA helicase is the key regulated step during DNA replication termination in eukaryotes, involving ubiquitylation of the Mcm7 helicase subunit, leading to a disassembly process that requires the Cdc48 "segregase". Here, we employ a screen to identify partners of budding yeast Cdc48 that are important for disassembly of ubiquitylated CMG helicase at the end of chromosome replication. We demonstrate that the ubiquitin-binding Ufd1-Npl4 complex recruits Cdc48 to ubiquitylated CMG. Ubiquitylation of CMG in yeast cell extracts is dependent upon lysine 29 of Mcm7, which is the only detectable site of ubiquitylation both in vitro and in vivo (though in vivo other sites can be modified when K29 is mutated). Mutation of K29 abrogates in vitro recruitment of Ufd1-Npl4-Cdc48 to the CMG helicase, supporting a model whereby Ufd1-Npl4 recruits Cdc48 to ubiquitylated CMG at the end of chromosome replication, thereby driving the disassembly reaction.**

## INTRODUCTION

Regulated unwinding of the parental DNA duplex ensures that eukaryotic cells make a single copy of their chromosomes during each cell cycle (Bell and Labib, 2016; Diffley, 2010). The essential DNA helicase at replication forks is built from 11 proteins (Moyer et al., 2006), which are assembled during G1 phase and S phase in a stepwise fashion at origins of DNA replication (Gambus et al., 2006), in a process that cannot be repeated until after cell division. The six Mcm2-7 ATPases form the catalytic core of the helicase and are loaded as a double hexamer around origin DNA during G1 phase (Deegan and Diffley, 2016). When cells enter S phase, the Cdc45 protein and the four-protein GINS complex are recruited to the Mcm2–7 core, leading to the formation of two replication forks per origin, each with an active CMG helicase (Gambus et al., 2006; Ilves et al., 2010; Pacek et al., 2006). To allow complete duplication of the genome, the CMG helicase must remain continuously associated with replication forks (Labib et al., 2000). However, the convergence of opposing replication forks leads to termination of DNA synthesis and to disassembly of the CMG helicase in a regulated process (Dewar et al., 2015; Maric et al., 2014; Moreno et al., 2014), the details of which are only beginning to emerge.

In budding yeast, the E3 ubiquitin ligase SCF$^{Dia2}$ is required to ubiquitylate CMG at the end of chromosome replication (Maculins et al., 2015; Maric et al., 2014). Ubiquitylated CMG is normally disassembled so quickly that it is undetectable in wild-type cells, but it can be stabilized by inactivation of Cdc48 (Maric et al., 2014), which is required to disrupt the ubiquitylated helicase into its component parts, namely Cdc45, Mcm2–7, and GINS. Work with extracts of *Xenopus laevis* eggs also demonstrated a role for p97/Cdc48 in release of ubiquitylated CMG from chromatin at the end of chromosome replication (Fullbright et al., 2016; Moreno et al., 2014; Semlow et al., 2016), indicating that the principal features of DNA replication termination have been conserved throughout the course of eukaryotic evolution.

Cdc48 has many ubiquitin-binding partners that are thought to recruit the segregase to a wide range of different substrates (Meyer et al., 2012). Here, we describe a screen in budding yeast for partners of Cdc48 that are required for helicase disassembly at the end of DNA replication. Our data indicate that the Ufd1-Npl4 heterodimer recruits Cdc48 to ubiquitylated CMG at the end of DNA synthesis, leading to a revised model for the final stages of eukaryotic chromosome duplication.

## RESULTS

### Ufd1-Npl4 Promote Disassembly of Ubiquitylated CMG Helicase

To screen systematically for Cdc48 partners that are required for CMG disassembly in vivo, we assayed for persistence of the ubiquitylated CMG helicase in budding yeast cells lacking each factor, using high salt extracts that prevented in vitro ubiquitylation of CMG by SCF$^{Dia2}$ (Maric et al., 2014). Removal of the non-essential partners of Cdc48 did not lead to persistence of ubiquitylated CMG (Figure S1A; CMG was isolated by immunoprecipitation of the Sld5 component of GINS) or to persistence of


This is an open access article under the CC BY license (http://creativecommons.org/licenses/by/4.0/).

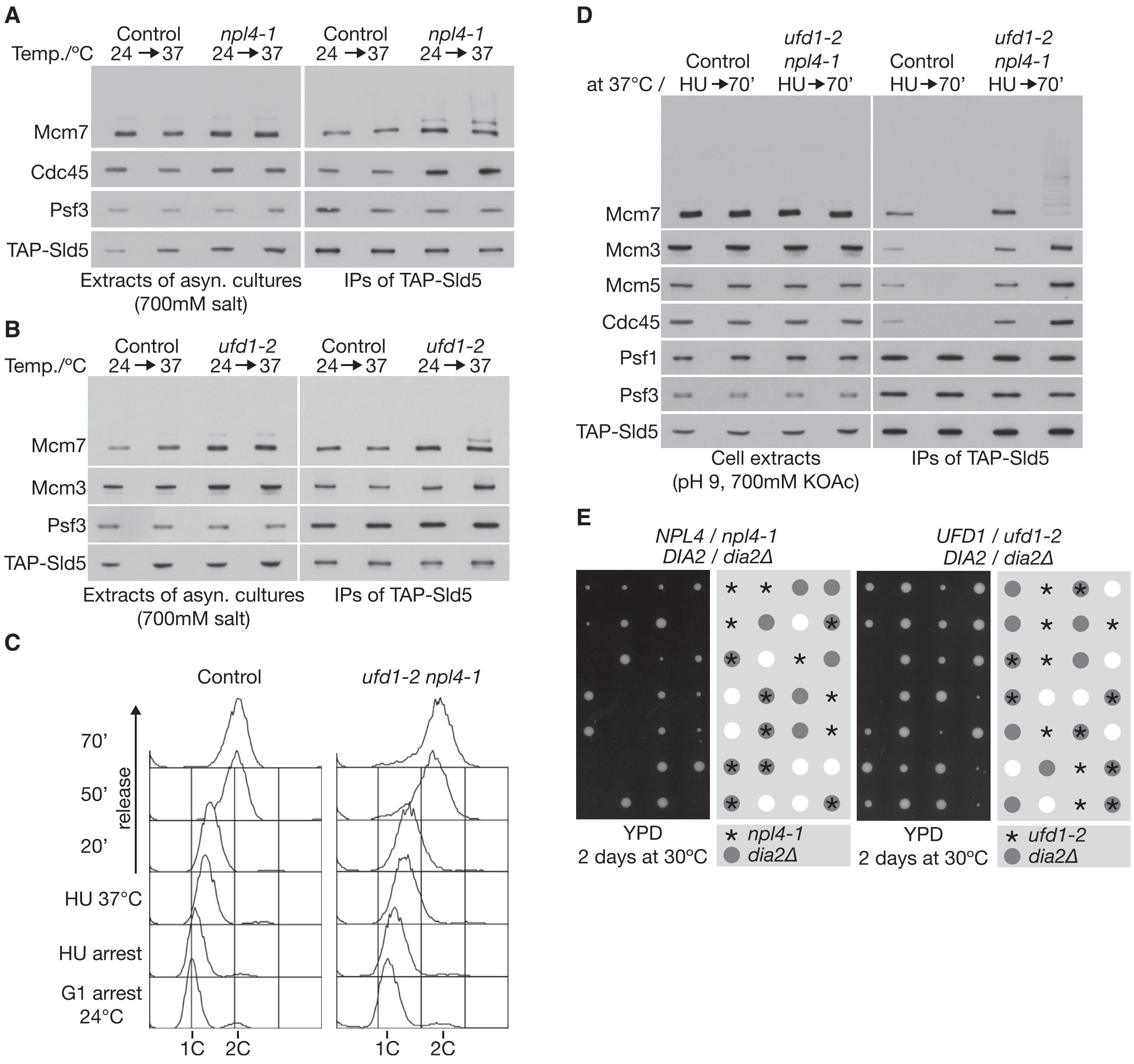


**Figure 1. Ufd1-Npl4 Are Required for Disassembly of the CMG Helicase at the End of Chromosome Replication in Budding Yeast**

(A) Asynchronous cultures of control cells (YASD375) and *npl4-1* (YMM308) were grown at 24°C and then shifted to 37°C for 1 hr before making cell extracts in the presence of 700 mM potassium acetate, in order to block in vitro ubiquitylation of CMG. The Sld5 subunit of GINS was then isolated by immunoprecipitation and the association of other CMG components monitored by immunoblotting. In vivo ubiquitylation of the Mcm7 subunit of CMG was observed in the *npl4-1* strain.

(B) A similar experiment to that above was performed with control cells (YASD375) and *ufd1-2* (YMM306).

(C) Control cells (YSS47) and *ufd1-2 npl4-1* cells (YMM574) were synchronized in G1 phase at 24°C and then released into S phase in the presence of 0.2 M hydroxyurea for 70 min to allow for CMG assembly at forks from early origins of replication. The cells were then shifted to 37°C for 60 min to inactivate Ufd1-Npl4, before washing into fresh medium lacking hydroxyurea, to allow cells to complete chromosome replication. Mating pheromone was added 45 min after release to prevent dividing cells from entering another round of S phase. DNA content was monitored by flow cytometry throughout the experiment, as shown in the panels.

(D) CMG was monitored as above by immunoprecipitation of the Sld5 subunit of GINS from high-salt extracts. Inactivation of Ufd1-Npl4 blocked CMG disassembly at the end of replication, leading to accumulation of CMG with in vivo ubiquitylated Mcm7.

(E) The indicated diploid strains (YMM562 and YMM563) were sporulated and the meiotic progeny dissected before growth for 2 days at 30°C. The genotype of each colony was then determined by replica plating to selective medium or to rich medium at 37°C.

See also Figures S1 and S2.

"old" CMG complexes from one cell cycle to the next (Figure S1B). The same was true after conditional depletion of degron-tagged Shp1 (Figures S1C and S1D), which couples Cdc48 to a variety of essential processes, including mitosis (Cheng and Chen, 2010). In contrast, ubiquitylation of the CMG helicase on its Mcm7 subunit was detected in asynchronous cultures of cells with temperature-sensitive mutations in either component of the essential Ufd1-Npl4 heterodimer (Figures 1A and 1B). Ufd1-Npl4 were previously shown to be required for degradation of Cdc48 substrates in the endoplasmic reticulum (Jentsch and Rumpf, 2007) but are also critical for extraction of ubiquitylated RNA polymerase II from chromatin in budding

yeast (Verma et al., 2011), as well as being important for the DNA damage response in both yeast and higher eukaryotes (Acs et al., 2011; Bergink et al., 2013; Davis et al., 2012; Meerang et al., 2011; Mosbech et al., 2012; Nie et al., 2012; Raman et al., 2011; Vaz et al., 2013).

Whereas CMG disassembly was partially defective when synchronized cultures of *ufd1-2* or *npl4-1* cells completed S phase at the restrictive temperature of 37°C, simultaneous inactivation of both factors in early S phase blocked the subsequent disassembly of CMG very efficiently (Figures S2A and S2B; note that *ufd1-2* cells showed a greater defect in CMG disassembly under these conditions than *npl4-1*). Correspondingly, inactivation of the Ufd1-Npl4 heterodimer led to accumulation of the CMG helicase with ubiquitylated Mcm7 subunit (Figures 1D and S2C), as seen previously upon inactivation of Cdc48 (Maric et al., 2014). These findings indicate that the Ufd1-Npl4 heterodimer is essential for disassembly of ubiquitylated CMG helicase at the end of chromosome replication in budding yeast. Consistent with this idea, the *npl4-1* mutation is synthetic lethal at a semi-permissive temperature with *dia2Δ* (Figure 1E), mirroring the synthetic lethality of *cdc48* alleles with *dia2* mutations (Maculins et al., 2015). Moreover, *ufd1-2* is synthetic sick at a semi-permissive temperature with *dia2Δ* (Figure 1E). In contrast, deletion of genes encoding the other Cdc48 adaptors is not lethal in combination with *dia2Δ* (M.M. and K.L., unpublished data).

## Ufd1-Npl4 Are Required to Recruit Cdc48 to the CMG Helicase

To establish that the Ufd1-Npl4-Cdc48 complex plays a direct role in CMG disassembly, we examined the association of all three factors with the helicase, using an in vitro system for SCF$^{Dia2}$-dependent ubiquitylation of CMG in yeast cell extracts (Maric et al., 2014). Whereas Cdc48-Ufd1-Npl4 did not associate with the GINS complex in extracts of G1 phase cells that lacked CMG, all three factors co-purified with GINS from S phase cell extracts (Figures 2A and 2B), together with the other components of the active helicase. We then repeated the same experiment using the *ufd1-2 npl4-1* double mutant in parallel with a control strain but first grew cells at the permissive temperature of 24°C and synchronized them in G1 phase before raising the temperature to 37°C and allowing cells to enter S phase (Figure 2C). As described above, Cdc48-Ufd1-Npl4 co-purified with the helicase from S phase extracts of control cells, but all three proteins failed to associate with CMG in extracts of the *ufd1-2 npl4-1* mutant (Figure 2D; Figures S2D and S2E show that the *ufd1-2* mutation makes the major contribution to this phenotype). These data indicate that the Ufd1-Npl4 heterodimer is required for recruitment of Cdc48 to the CMG helicase in budding yeast.

## CMG Is Ubiquitylated on a Single Lysine in the Mcm7 Subunit

Ufd1 and Npl4 both contain ubiquitin-binding modules (Meyer et al., 2002; Park et al., 2005; Wang et al., 2003), leading us to investigate the role of Mcm7 ubiquitylation in Ufd1-Npl4-dependent recruitment of Cdc48 to the CMG helicase. To try and map the sites of SCF$^{Dia2}$-dependent ubiquitylation by mass spectrometry, we first developed a strategy for enrichment of ubiquitylated Mcm7 from the isolated CMG complex after in vitro ubiquitylation of the helicase in extracts of S phase cells (see Experimental Procedures). Initially, however, repeated attempts to map the ubiquitylation sites in trypsin-digested Mcm7 were unsuccessful, despite over 90% coverage of the peptide sequence. Therefore, we took an alternative approach by mutating clusters of surface lysine residues (Figure 3A), predicted by comparison with crystal structures of archaeal MCM proteins (Brewster et al., 2008). The mutated Mcm7 proteins (or wild-type Mcm7) were expressed in *mcm7-td* cells (Figure 3B), in which the endogenous *MCM7* gene was tagged with the heat-inducible degron (Dohmen et al., 1994), to allow rapid degradation at 37°C in the presence of the E3 ubiquitin ligase Ubr1 (Labib et al., 2000). Cells were arrested at 24°C in G2/M phase with nocodazole before induced expression of Ubr1 and mutated/wild-type Mcm7 from the *GAL1* promoter, followed by degradation of Mcm7-td at 37°C. The cells were subsequently allowed to divide, arrested transiently in the subsequent G1 phase, and then finally released into S phase (Figure 3C) before isolation of the GINS component of the CMG helicase from cell extracts.

Whereas degradation of Mcm7-td was sufficient to block CMG assembly in the subsequent S phase (Figure 3D, sample 1), this defect could be rescued by expression of wild-type Mcm7 (Figure 3D, sample 2). Moreover, CMG complexes containing wild-type Mcm7 were ubiquitylated efficiently in the yeast cell extracts (Figure 3D, sample 2). Ubiquitylation was not affected by mutating clusters of predicted surface lysines in the C-terminal half of Mcm7 (data summarized in Figure 3A). In contrast, mutation of the first seven lysines of Mcm7 blocked in vitro ubiquitylation, indicating that the modification was restricted to the amino terminus of the Mcm7 protein. Strikingly, subsequent deconvolution experiments (summarized in Figure 3A) showed that the first lysine of Mcm7 was essential for the observed in vitro ubiquitylation (Figures 3C and 3D, sample 3, *mcm7-K29A*).

Lysine 29 is located within the longest tryptic peptide of Mcm7, probably contributing to the initial difficulty in detecting the sites of Mcm7 ubiquitylation by mass spectrometry. Therefore, we combined trypsin with the endoproteinase GluC that cuts after glutamate residues to reduce the size of the cleaved peptide containing K29. After partial purification of Mcm7 from isolated CMG helicase that had been ubiquitylated in yeast cell extracts (Figure S3A), subsequent analysis of peptides cleaved with trypsin and GluC revealed a single site of in vitro ubiquitylation of Mcm7, namely K29 (Figure S3C; Table S1). Moreover, K29 was also the only detectable site of in vivo ubiquitylation of Mcm7 when we purified the CMG helicase from high salt cell extracts after inactivation of Cdc48 to block the disassembly of ubiquitylated CMG at the end of S phase (Figures S3B and S3D; Table S1). These experiments indicate that SCF$^{Dia2}$-dependent ubiquitylation of the 11-subunit CMG helicase is remarkably specific both in vivo and in yeast cell extracts.

## CMG Ubiquitylation Promotes Recruitment of Ufd1-Npl4-Cdc48

Mapping and mutating the site of Mcm7 modification allowed us to explore in vitro the role of ubiquitylation in recruitment of

OPEN
ACCESS
CellPress

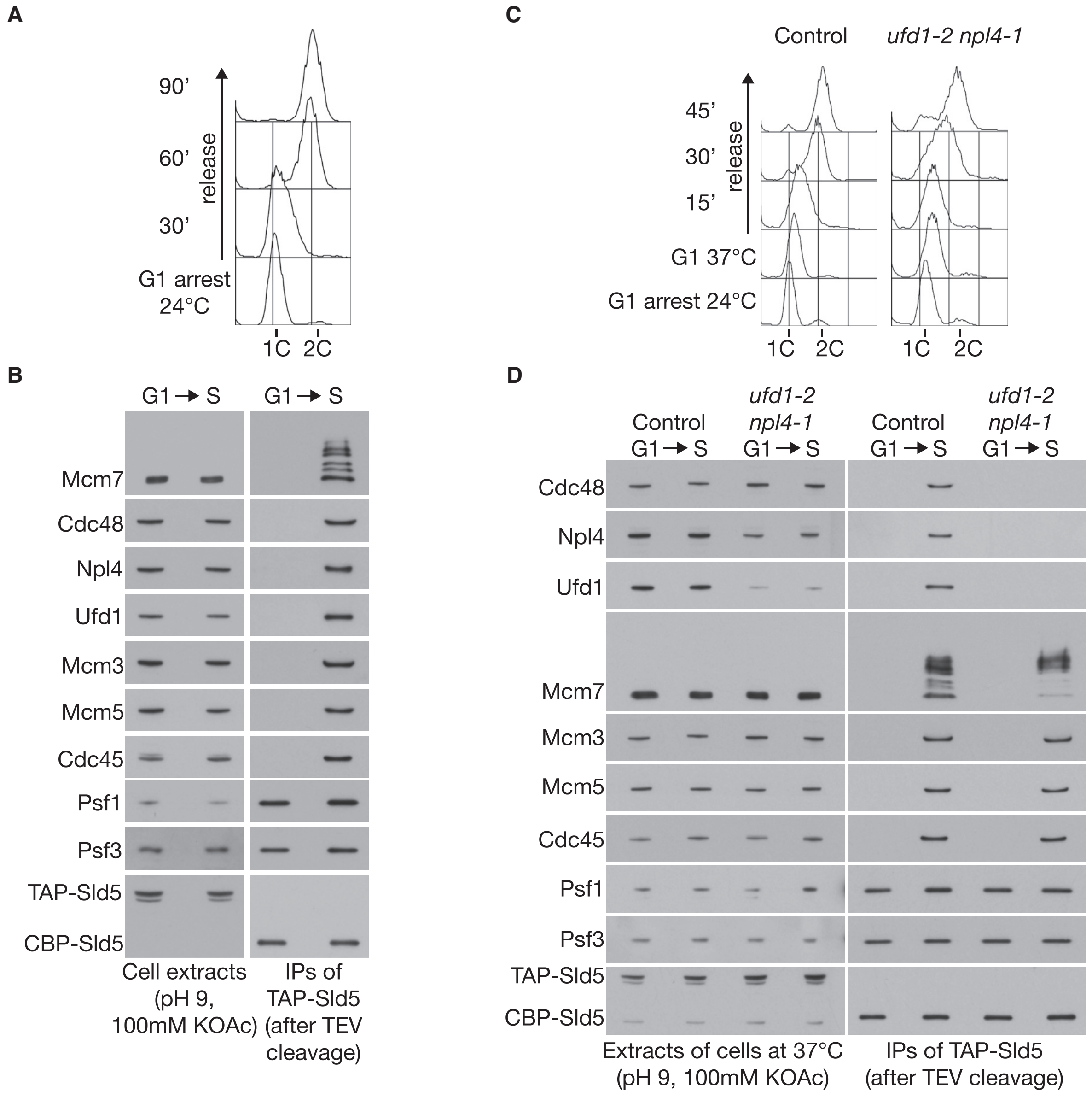


**Figure 2. Ufd1-Npl4 Are Required to Recruit Cdc48 to the CMG Helicase**

(A) *TAP-SLD5* cells (YSS47) were synchronized in G1 phase at 24°C and then released into S phase for the indicated times. DNA content was measured by flow cytometry.

(B) The association of Ufd1, Npl4, and Cdc48 with CMG was monitored by immunoprecipitation of GINS from G1 phase or S phase cells (30 min time point) under conditions that favor SCF$^{Dia2}$-dependent in vitro ubiquitylation of the helicase.

(C) Control (YSS47) and *ufd1-2 npl4-1* cells (YMM574) were synchronized in G1 phase at 24°C and then shifted to 37°C for 60 min before release into S phase for 15 min.

(D) The association of Ufd1, Npl4, and Cdc48 with CMG was monitored as above.

Ufd1-Npl4-Cdc48 to the CMG helicase. We generated haploid yeast strains in which the endogenous *MCM7* locus was modified in order to introduce the *mcm7-K29A* or *mcm7-K29R* mutations (see Experimental Procedures). In extracts of synchronized S phase cell cultures, we found that either mutation abolished in vitro ubiquitylation of the CMG helicase, compared to control cell extracts (Figures 4A, 4B, S4A, and S4B). Whereas Ufd1-Npl4-Cdc48 associated with the ubiquitylated CMG helicase in control cell extracts as described above (Figure 4B, *MCM7*), this association was greatly diminished when in vitro ubiquitylation of CMG was blocked by mutation of K29 of Mcm7 (Figure 4B, *mcm7-K29A*). These findings indicate that ubiquitylation of Mcm7 plays an important role in recruiting Ufd1-Npl4-Cdc48 to the CMG helicase.

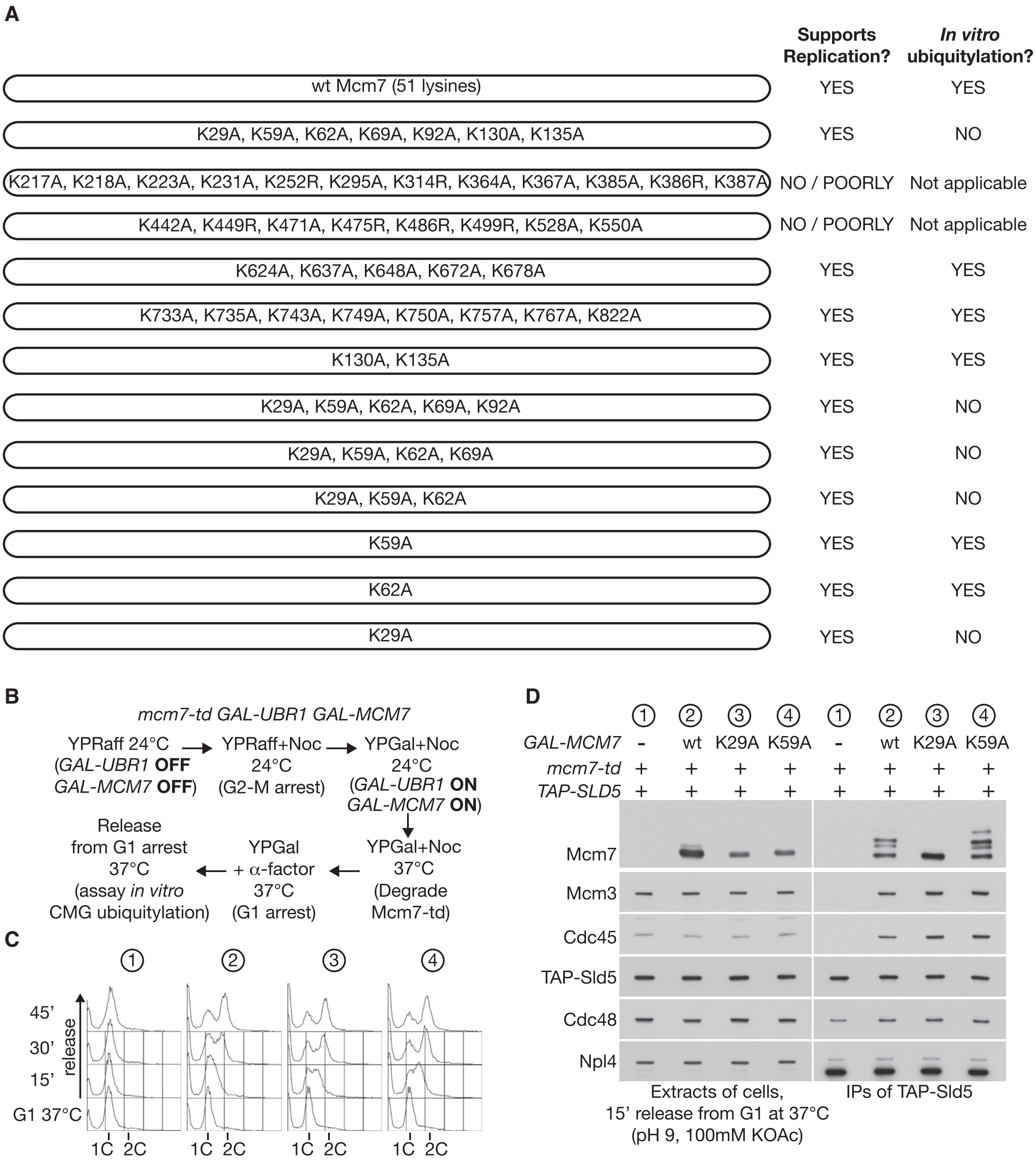


**Figure 3. In Vitro Ubiquitylation of CMG in Yeast Cell Extracts Is Dependent upon Lysine 29 of Mcm7**

(A) Summary of screen for alleles of Mcm7 that cannot be ubiquitylated in vitro.
(B) Experimental scheme.
(C) DNA content was monitored by flow cytometry at the indicated time points.
(D) CMG was monitored as above.
See also Figure S3 and Table S1.

## In Vivo Ubiquitylation of Mcm7-K29A during Termination

Lysine 29 of Mcm7 is the only detectable site of CMG ubiquitylation in wild-type yeast cells both in vivo and in vitro (Figure S3), and mutation of K29 abolishes in vitro CMG ubiquitylation in yeast cell extracts (Figures 3D and 4B). Nevertheless, we found that another site (or other sites) on Mcm7 can be ubiquitylated in vivo when *mcm7-K29A* cells complete S phase (Figures S4C–S4E, *mcm7-K29A*, sample 2). Moreover, in vivo ubiquitylation of Mcm7-K29A occurs with comparable efficiency to CMG ubiquitylation on lysine 29 of Mcm7 in wild-type cells (Figure S4E,

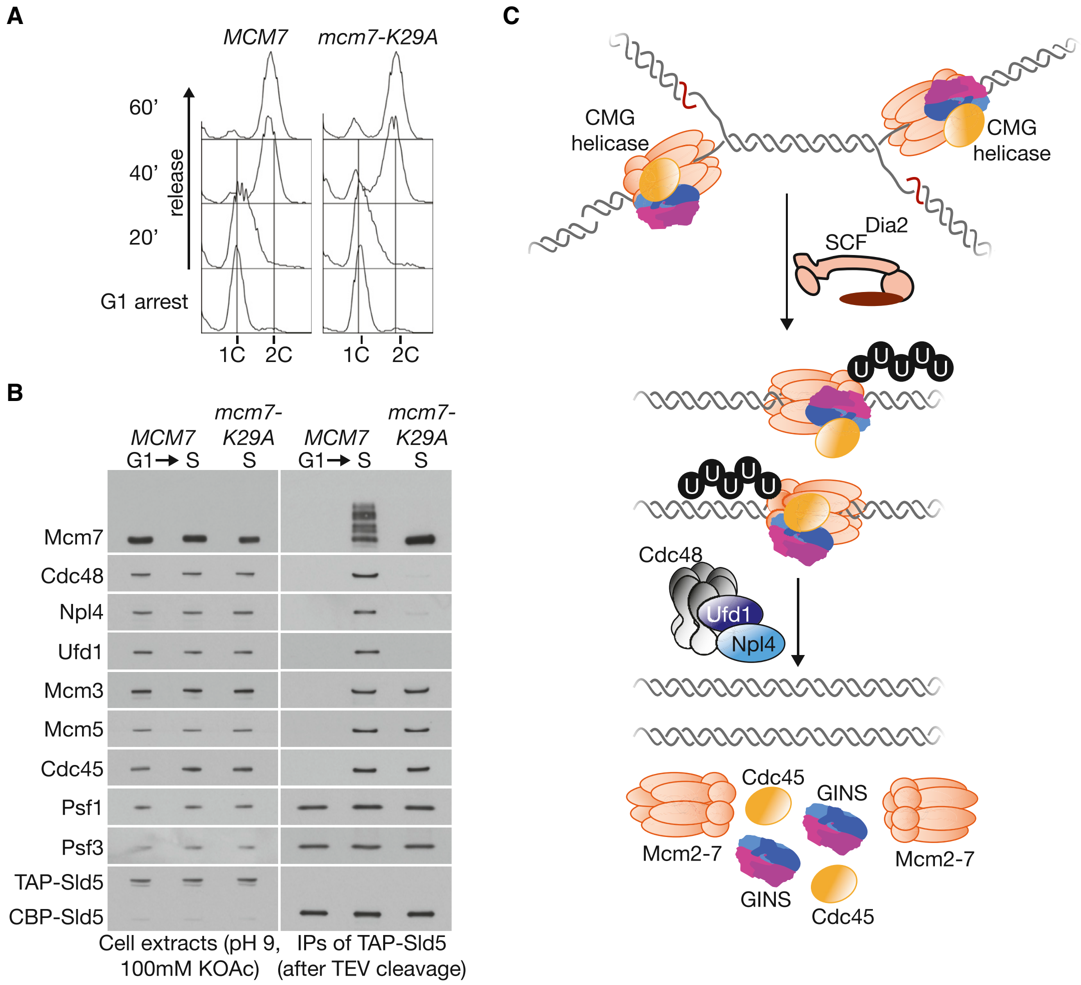


**Figure 4. Ubiquitylation of the Mcm7 Subunit of CMG Drives Recruitment of Ufd1-Npl4-Cdc48**

(A) Control (YGDP483) and *mcm7-K29A* (YMM493) yeast cells were synchronized in G1 phase at 30°C and then released into S phase for 20 min.

(B) The association of Ufd1, Npl4, and Cdc48 with CMG was monitored as above.

(C) Model for CMG disassembly at the end of chromosome replication in budding yeast. Note that the fate of ubiquitylated Mcm7 after CMG disassembly is unknown.

See also Figure S4.

sample 2; compare *MCM7* with *mcm7-K29A*). Interestingly, therefore, the regulation of CMG ubiquitylation ensures that it is highly specific in wild-type yeast cells, being limited to a single lysine residue on Mcm7, and yet is still able to switch elsewhere on Mcm7 with high efficiency when K29 of Mcm7 is mutated (note that we cannot exclude the alternative possibility that Mcm7 in wild-type cells is ubiquitylated in vivo, but not in vitro, on another site that is not detected in our mass spectrometry assays, in addition to ubiquitylation of K29). Ubiquitylation of CMG in yeast cell extracts is more constrained, at least under our in vitro conditions, such that K29 of Mcm7 is the only possible acceptor site for ubiquitin.

In vivo ubiquitylation of Mcm7-K29A on the alternative site(s) appears to be functionally important during DNA replication termination, because CMG disassembly is highly efficient when *mcm7-K29A* cells complete S phase (Figures S4F and S4G). As would be expected, therefore, *mcm7-K29A* cells grow like a wild-type strain and lack phenotypes of *dia2Δ* cells (Morohashi et al., 2009), such as synthetic lethality upon deletion of the gene encoding the Rrm3 DNA helicase (Figure S4H).

## DISCUSSION

Our data lead to a revised model for CMG disassembly in budding yeast, beginning with the addition of a K48-linked ubiquitin chain (Maric et al., 2014) to the Mcm7 subunit of the helicase at the end of chromosome replication, dependent upon $SCF^{Dia2}$ (Figure 4C). Ubiquitylation of CMG then drives recruitment of Cdc48, via the Ufd1-Npl4 heterodimer. Finally, the Cdc48 segregase disassembles the CMG helicase and releases its component parts from chromatin. The role of the Ufd1-Npl4 heterodimer in CMG disassembly is likely to be conserved in all eukaryotes. Previous work showed that depletion of the orthologues of Ufd1 or Npl4 led to persistence of Cdc45 or GINS on mitotic chromatin in early

embryos of the nematode *Caenorhabditis elegans* or in egg extracts of the frog *Xenopus laevis* (Franz et al., 2011). Although these findings were thought to reflect a post-replicative role for UFD1-NPL4-CDC48 in extracting GINS and Cdc45 (but not Mcm2–7) from chromatin during mitosis, we found that depletion of UFD-1, NPL-4, or CDC-48 in *C. elegans* early embryos leads to persistence of ubiquitylated CMG helicase (Sonneville et al., 2017). Moreover, UFD1-NPL4-p97 are recruited to chromatin during DNA replication termination in frog egg extracts (Dewar et al., 2017) and associate with ubiquitylated CMG helicase (Sonneville et al., 2017). These data indicate a conserved mechanism for CMG disassembly from yeast to animals.

The triggers for CMG ubiquitylation during the termination of replication remain to be defined. It is possible that the helicases at converged forks slide onto double-stranded DNA (dsDNA) when they meet the 5′ end of the opposing fork's lagging strand, as CMG disassembly has been shown to occur after all DNA intermediates have been ligated and DNA synthesis is complete (Dewar et al., 2015). The change from encircling single-stranded DNA (ssDNA) to dsDNA might provide a signal for ubiquitylation (Dewar et al., 2015), as might the detachment of the helicase from DNA at telomeres, where termination only involves a single fork. We suggest that such changes in helicase action at the end of replication produce a structural change in CMG that favors ubiquitylation of the amino terminal site in Mcm7. In this context, it is interesting to note that lysine 29 of Mcm7 is located within "sub-domain A," which in archaeal MCM helicases undergoes a 150° rotation between different structural forms (Chen et al., 2005; Miller et al., 2014). More recently, electron microscopy structures of yeast Mcm2–7 (Li et al., 2015) and *Drosophila* CMG (Abid Ali et al., 2016) indicate that sub-domain A of Mcm7 rotates significantly between the loaded inactive Mcm2–7 complex and the active CMG helicase. It thus seems plausible that structural changes in sub-domain A of Mcm7 might represent a conformational switch on the CMG helicase during termination, for example, revealing a docking site for the ubiquitin ligase or otherwise controlling the access of the ligase to K29. Ubiquitylation of Mcm7 then promotes recruitment of Ufd1-Npl4 and thus of Cdc48, leading locally to helicase disassembly after DNA synthesis has been completed in each particular replicon.

It remains to be determined how ubiquitylation of CMG is restricted to a specific lysine residue in wild-type cells, and yet is able to switch in vivo (but not in cell extracts) to other sites upon mutation of lysine 29 of Mcm7. It will be important in future studies to map and mutate the alternative sites of ubiquitylation of Mcm7-K29A. The ultimate goal would be to create a non-ubiquitylatable version of the CMG helicase, in order to test directly the significance of CMG ubiquitylation for helicase disassembly at the end of chromosome replication and explore the importance of CMG ubiquitylation for genome integrity in yeast cells that still contain active $SCF^{Dia2}$.

## EXPERIMENTAL PROCEDURES

### Yeast Strains

Table S2 lists the yeast strains used in this study. All genetic modifications were initially made in the diploid budding-yeast strain W303-1. Following confirmation of the genome modification by a series of PCR reactions, the haploid strains used for subsequent analyses were obtained by tetrad dissection.

The *TET-shp1-aid* allele was constructed using a modified version of the auxin-inducible degron system (Nishimura et al., 2009; Tanaka et al., 2015). First, the tetracycline-repressible promotor was introduced at the 5′ end of the *SHP1* gene in diploid cells expressing the rice Tir1 E3 ubiquitin ligase. Subsequently, the "3× mini auxin-inducible degron" cassette was introduced at the 3′ end of the *SHP1* coding sequence.

A two-step strategy was used to introduce the *mcm7-K29A* or *mcm7-K29R* mutations into the endogenous *MCM7* locus in budding yeast cells. First, the region encoding amino acids 1–64 of Mcm7 was replaced with the URA3 marker, in one copy of *MCM7* in diploid cells, to produce *MCM7/mcm7Δ::URA3*. Second, the entire *MCM7* locus was PCR amplified with flanking sequences from *MCM7-5FLAG9His* cells, in which the *hphNT* hygromycin-resistance marker is located after the tag at the end of MCM7 coding sequence. The resulting fragment was cloned into pBluescript II KS+, and site-directed mutagenesis was used to introduce the K29A or K29R mutations. The resulting fragments were then excised and transformed into the heterozygous *MCM7/mcm7Δ::URA3* diploid cells. Selection for the *hphNT* marker and confirmation of loss of *URA3* led to the isolation of *MCM7/mcm7-K29A-5FLAG9His::hphNT* and *MCM7/mcm7-K29R-5FLAG9His::hphNT* diploid strains. The *mcm7-K29A-5FLAG9His::hphNT* and *mcm7-K29R-5FLAG9His::hphNT* haploid strains were then derived by sporulation and tetrad dissection of the corresponding diploids before final confirmation of the mutations by DNA sequencing.

The temperature-sensitive strains *ufd1-2* and *npl4-1* were a generous gift from Raymond Deshaies and, before use, were backcrossed eight times with our wild-type W303 strains.

### Growing Yeast Liquid Cultures

Unless otherwise stated, budding yeast cells were grown in standard YP medium (1% yeast extract; 2% peptone) containing 2% glucose (YPD). For the cultures involving *mcm7-td* strains (Figure 3), cells were inoculated at 24°C the previous day in YP supplemented with 2% raffinose (YPRaff) and 0.1 mM copper (II) sulfate and then diluted in the same medium the next morning before growth of a mid-log culture. Expression of the Ubr1 E3 ubiquitin ligase was then induced by switching the medium to YP supplemented with 2% galactose (YPGal), first at 24°C for 30 min and subsequently at 37°C for 60 min.

Other yeast cultures were grown at 24°C or 30°C. In order to arrest cells in the G1 phase of the cell cycle, the mating pheromone alpha factor (Pepceuticals) was initially added to cultures at 7.5 μg/mL and then supplemented by an additional 2.5 μg/mL every 20 min after 60 min from initial incubation (for cultures grown at 30°C or every 30 min after 90 min if the cultures were grown at 24°C) until 90% or more of cells formed "shmoos". For *dia2Δ* cells, as well as for *ufd1-2* and *npl4-1* cells, the additional supplements of alpha factor were increased to 5 μg/mL.

For the experiments shown in Figure 3, 5 μg/mL nocodazole was used to arrest cells in the G2/M phase of the cell cycle. After induction of *GAL-UBR1* in YPGal medium, cells were washed into YPGal medium containing 10 μg/mL alpha factor, and additional aliquots of 2.5 μg/mL alpha factor were then added after 30 min and then again every 15 min.

To arrest cells in early S phase, cells were grown in the presence of 0.2 M hydroxyurea (HU).

Degradation of the Shp1-aid protein (in the experiment shown in Figure S1D) was induced by the addition for 3 hr of auxins (1 mM indole-3-acetic acid and 0.5 mM 1-napthaleneacetic acid) and 20 μg/mL doxycycline to an asynchronous mid-log culture at 30°C, which was grown overnight in the presence of 0.25 μg/mL doxycycline.

### Tetrad Dissection

For the experiments shown in Figures 1E and S4H, *dia2Δ* cells were mated with cells with deletions of Cdc48 cofactors or to cells with the temperature-sensitive mutations *ufd1-2* and *npl4-1* at 30°C due to the cold sensitivity of *dia2Δ* cells. Sporulated diploids were then dissected and plates were

OPEN
ACCESS
CellPress

incubated at 30°C for 2 days. The genotype of the haploid progeny was determined by replica plating to selective media.

### Dilution Spotting

For the experiment shown in Figure S1C, cells were diluted to $3 \times 10^6$, $3 \times 10^5$, $3 \times 10^4$, and $3 \times 10^3$ cells/mL in PBS. Spots containing $50 \times 10^4$, $50 \times 10^3$, $50 \times 10^2$, or 50 cells were then placed on the indicated media (YPD or YPD + 0.5 mM IAA + 0.5 mM NAA + 20 μg/mL doxycycline).

### Flow Cytometry

The content of DNA in cells was monitored by flow cytometry of fixed cells stained with propidium iodide (Labib et al., 2000). Flow cytometers used in this study were FACSCalibur and FACSCanto II (Becton Dickinson). The measurements were analyzed with FlowJo software (Tree Star).

### Immunoprecipitation of Protein Complexes from Yeast Extracts

The isolation of protein complexes from budding yeast cell extracts was performed as described previously (Maric et al., 2014). In the experiments shown in Figures 1C, 1D, 2, S1, S2B, S3B, S3D, S4A, and S4B, the deubiquitylase inhibitor propargylated ubiquitin (Ub-Prg, supplied by the Protein Production Team of the MRC PPU at the University of Dundee) was added to cell extracts to a final concentration of 5μM. For in vitro ubiquitylation experiments, Ub-Prg was added to low salt yeast cell extracts upon thawing, whereas, for in vivo ubiquitylation experiments, the Ubi-Prg was first added to the "high-salt lysis buffer," in which cells were frozen and then Ub-Prg was also added to the thawed cell extracts as above.

### Isolation of Ubiquitylated Mcm7 for Mass Spectrometry Analysis

After in vitro ubiquitylation in low-salt extracts (100 mM potassium acetate) or in vivo ubiquitylation after inactivation of Cdc48-aid (using high salt extracts with 700 mM potassium acetate to block in vitro ubiquitylation plus 5 μM Ub-Prg to inhibit deubiquitylases), we isolated the Mcm7 subunit of the CMG helicase using the two-step purification procedure that we described previously (Maric et al., 2014).

### Mass Spectrometry and Data Analysis

Peptides were extracted using the in-gel tryptic digestion protocol described previously (Shevchenko et al., 2006). The extracted tryptic peptides were then resuspended in 26 μL 0.5% acetic acid, 0.1% trifluoroacetic acid (TFA) ready for mass spectrometry analysis or for further digestion with Glu-C. Peptides from approximately half of this volume were purified using "stop and go extraction tips" (Rappsilber et al., 2003) and then resuspended in 20 μL of 50 mM ammonium bicarbonate containing 0.2 μg endoproteinase Glu-C (sequencing grade; Roche) before incubation for 16 hr at room temperature. The Glu-C digestions were dried down using a vacuum centrifuge at 30°C, and the resultant doubly digested peptides were resuspended in 20 μL of 0.5% acetic acid and 0.1% TFA ready for mass spectrometry analysis. In this way, two peptide samples were generated for each slice of gel and digested either just with trypsin or else with trypsin + Glu-C.

Mass spectrometry analysis was performed by liquid chromatography-tandem mass spectrometry (LC-MS/MS) (Q Exactive; Thermo Fisher Scientific) equipped with an integrated nano-electrospray (Easy-Spray; Thermo Fisher Scientific) coupled to a nano-uHPLC system (Easy N-LC-1000; Thermo Fisher Scientific). Peptides were fractionated on a 50 cm × 75 μm internal diameter, PepMap RSLC C18, 2 μm reverse-phase column heated to 45°C. For initial analysis, between 2 and 6 μL peptide samples were loaded and fractionated over a 60-min gradient. Data were acquired in the data-dependent mode. Full scan spectra (*m/z* 300–1,800) were acquired in the Orbitrap with resolution $R$ = 70,000 at *m/z* 200 (after accumulation to a target value of 1,000,000 or 20 ms). The ten most intense ions were fragmented by "higher energy collisional dissociation" (resolution 17,500 at *m/z* 200 and target value of 500,000 or 60 ms). Precursor ions of unassigned charge, +1, or >+8 charge were excluded from the MS/MS analysis. The intensity threshold was set to $2 \times 10^4$. To obtain higher quality spectra of GlyGly-K-containing peptides, further MS runs were performed on the doubly digested peptides (trypsin + GluC) using the same parameters as described above, with the exception of selecting only the top seven peptides for MS/MS analysis at a resolution of 35,000, with a target value of 1,000,000 ions or a fill time of 150 ms.

All the raw MS data files were analyzed together using the quantitative MS software MaxQuant (Cox and Mann, 2008) incorporating the Andromeda search engine (version 1.5.2.8; Cox et al., 2011). An *S. cerevisiae* protein database was searched along with the exact sequence of the Mcm7-5FLAG-9His construct. Enzyme specificity was set to trypsin-P or trypsin-P + Glu-C as required. Cysteine carbamidomethylation was selected as a fixed modification and GlyGly-lysine, methionine oxidation, and protein N-acetylation were searched as variable modifications. The initial maximum allowed mass deviation was set to 20 parts per million (ppm) for peptide masses and 0.5 Da for MS/MS peaks. The minimum peptide length was set to seven amino acids and a maximum of two missed cleavages (Trypsin-P) or three missed cleavages (Trypsin-P + Glu-C). A 1% false discovery rate (FDR) was required at both the protein and peptide level. In addition to the FDR threshold, proteins were considered as "identified," if they had at least one unique peptide. Protein identifications and intensity values based on extracted ion chromatograms were reported for each slice from each gel lane. Spectral annotations were performed automatically in MaxQuant.

### Immunoblotting

Polyclonal antibodies for the detection of replisome components and Cdc48 by immunoblotting were described previously (Foltman et al., 2013; Gambus et al., 2006, 2009; Maric et al., 2014). Polyclonal antibodies against full-length Ufd1 and Npl4 1-222 were produced by MRC PPU reagents (https://mrcppureagents.dundee.ac.uk) at the University of Dundee.

## SUPPLEMENTAL INFORMATION

Supplemental Information includes four figures and two tables and can be found with this article online at http://dx.doi.org/10.1016/j.celrep.2017.03.020.

## AUTHOR CONTRIBUTIONS

M.M. performed all experiments except for the following: P.M. performed the experiments in Figures S1D, 2D, 2E, and 4A–4G and also re-ran gels for Figures 1D and S2C and M.H.T. carried out the mass spectrometry analysis in Figure S3, with input from R.H. K.L. and M.M. conceived the project and designed experiments in collaboration with P.M., M.H.T., and R.H. K.L. wrote the manuscript, with contributions and critical comments from the other authors.

## ACKNOWLEDGMENTS

We gratefully acknowledge the support of the Medical Research Council (core grant MC_UU_12016/13), the Wellcome Trust (references 097945/B/11/Z for flow cytometry, 102943/Z/13/Z for an Investigator award to K.L., and 098391/Z/12/Z for an Investigator award to R.H.), and Cancer Research UK (reference C434/A13067 for an award to R.H.). We thank Axel Knebel, Clare Johnson, and Richard Ewan for producing propargylated ubiquitin; Melanie Wightman, Nikki Wood, and Mark Peggie for plasmids; MRC PPU Reagents and Services (https://mrcppureagents.dundee.ac.uk) for antibodies; and Raymond Deshaies, Masato Kanemaki, and Seiji Tanaka for strains.

Received: February 3, 2017
Revised: February 28, 2017
Accepted: March 3, 2017
Published: March 28, 2017

## REFERENCES

Abid Ali, F., Renault, L., Gannon, J., Gahlon, H.L., Kotecha, A., Zhou, J.C., Rueda, D., and Costa, A. (2016). Cryo-EM structures of the eukaryotic replicative helicase bound to a translocation substrate. Nat. Commun. *7*, 10708.

Acs, K., Luijsterburg, M.S., Ackermann, L., Salomons, F.A., Hoppe, T., and Dantuma, N.P. (2011). The AAA-ATPase VCP/p97 promotes 53BP1 recruitment by

removing L3MBTL1 from DNA double-strand breaks. Nat. Struct. Mol. Biol. *18*, 1345–1350.

Bell, S.P., and Labib, K. (2016). Chromosome duplication in Saccharomyces cerevisiae. Genetics *203*, 1027–1067.

Bergink, S., Ammon, T., Kern, M., Schermelleh, L., Leonhardt, H., and Jentsch, S. (2013). Role of Cdc48/p97 as a SUMO-targeted segregase curbing Rad51-Rad52 interaction. Nat. Cell Biol. *15*, 526–532.

Brewster, A.S., Wang, G., Yu, X., Greenleaf, W.B., Carazo, J.M., Tjajadi, M., Klein, M.G., and Chen, X.S. (2008). Crystal structure of a near-full-length archaeal MCM: functional insights for an AAA+ hexameric helicase. Proc. Natl. Acad. Sci. USA *105*, 20191–20196.

Chen, Y.J., Yu, X., Kasiviswanathan, R., Shin, J.H., Kelman, Z., and Egelman, E.H. (2005). Structural polymorphism of Methanothermobacter thermautotrophicus MCM. J. Mol. Biol. *346*, 389–394.

Cheng, Y.L., and Chen, R.H. (2010). The AAA-ATPase Cdc48 and cofactor Shp1 promote chromosome bi-orientation by balancing Aurora B activity. J. Cell Sci. *123*, 2025–2034.

Cox, J., and Mann, M. (2008). MaxQuant enables high peptide identification rates, individualized p.p.b.-range mass accuracies and proteome-wide protein quantification. Nat. Biotechnol. *26*, 1367–1372.

Cox, J., Neuhauser, N., Michalski, A., Scheltema, R.A., Olsen, J.V., and Mann, M. (2011). Andromeda: a peptide search engine integrated into the MaxQuant environment. J. Proteome Res. *10*, 1794–1805.

Davis, E.J., Lachaud, C., Appleton, P., Macartney, T.J., Näthke, I., and Rouse, J. (2012). DVC1 (C1orf124) recruits the p97 protein segregase to sites of DNA damage. Nat. Struct. Mol. Biol. *19*, 1093–1100.

Deegan, T.D., and Diffley, J.F. (2016). MCM: one ring to rule them all. Curr. Opin. Struct. Biol. *37*, 145–151.

Dewar, J.M., Budzowska, M., and Walter, J.C. (2015). The mechanism of DNA replication termination in vertebrates. Nature *525*, 345–350.

Dewar, J.M., Low, E., Mann, M., Räschle, M., and Walter, J.C. (2017). CRL2Lrr1 promotes unloading of the vertebrate replisome from chromatin during replication termination. Genes Dev. *31*, 275–290.

Diffley, J.F. (2010). The many faces of redundancy in DNA replication control. Cold Spring Harb. Symp. Quant. Biol. *75*, 135–142.

Dohmen, R.J., Wu, P., and Varshavsky, A. (1994). Heat-inducible degron: a method for constructing temperature-sensitive mutants. Science *263*, 1273–1276.

Foltman, M., Evrin, C., De Piccoli, G., Jones, R.C., Edmondson, R.D., Katou, Y., Nakato, R., Shirahige, K., and Labib, K. (2013). Eukaryotic replisome components cooperate to process histones during chromosome replication. Cell Rep. *3*, 892–904.

Franz, A., Orth, M., Pirson, P.A., Sonneville, R., Blow, J.J., Gartner, A., Stemmann, O., and Hoppe, T. (2011). CDC-48/p97 coordinates CDT-1 degradation with GINS chromatin dissociation to ensure faithful DNA replication. Mol. Cell *44*, 85–96.

Fullbright, G., Rycenga, H.B., Gruber, J.D., and Long, D.T. (2016). p97 promotes a conserved mechanism of helicase unloading during DNA cross-link repair. Mol. Cell. Biol. *36*, 2983–2994.

Gambus, A., Jones, R.C., Sanchez-Diaz, A., Kanemaki, M., van Deursen, F., Edmondson, R.D., and Labib, K. (2006). GINS maintains association of Cdc45 with MCM in replisome progression complexes at eukaryotic DNA replication forks. Nat. Cell Biol. *8*, 358–366.

Gambus, A., van Deursen, F., Polychronopoulos, D., Foltman, M., Jones, R.C., Edmondson, R.D., Calzada, A., and Labib, K. (2009). A key role for Ctf4 in coupling the MCM2-7 helicase to DNA polymerase alpha within the eukaryotic replisome. EMBO J. *28*, 2992–3004.

Ilves, I., Petojevic, T., Pesavento, J.J., and Botchan, M.R. (2010). Activation of the MCM2-7 helicase by association with Cdc45 and GINS proteins. Mol. Cell *37*, 247–258.

Jentsch, S., and Rumpf, S. (2007). Cdc48 (p97): a "molecular gearbox" in the ubiquitin pathway? Trends Biochem. Sci. *32*, 6–11.

Labib, K., Tercero, J.A., and Diffley, J.F.X. (2000). Uninterrupted MCM2-7 function required for DNA replication fork progression. Science *288*, 1643–1647.

Li, N., Zhai, Y., Zhang, Y., Li, W., Yang, M., Lei, J., Tye, B.K., and Gao, N. (2015). Structure of the eukaryotic MCM complex at 3.8 Å. Nature *524*, 186–191.

Maculins, T., Nkosi, P.J., Nishikawa, H., and Labib, K. (2015). Tethering of SCF(Dia2) to the replisome promotes efficient ubiquitylation and disassembly of the CMG helicase. Curr. Biol. *25*, 2254–2259.

Maric, M., Maculins, T., De Piccoli, G., and Labib, K. (2014). Cdc48 and a ubiquitin ligase drive disassembly of the CMG helicase at the end of DNA replication. Science *346*, 1253596.

Meerang, M., Ritz, D., Paliwal, S., Garajova, Z., Bosshard, M., Mailand, N., Janscak, P., Hübscher, U., Meyer, H., and Ramadan, K. (2011). The ubiquitin-selective segregase VCP/p97 orchestrates the response to DNA double-strand breaks. Nat. Cell Biol. *13*, 1376–1382.

Meyer, H.H., Wang, Y., and Warren, G. (2002). Direct binding of ubiquitin conjugates by the mammalian p97 adaptor complexes, p47 and Ufd1-Npl4. EMBO J. *21*, 5645–5652.

Meyer, H., Bug, M., and Bremer, S. (2012). Emerging functions of the VCP/p97 AAA-ATPase in the ubiquitin system. Nat. Cell Biol. *14*, 117–123.

Miller, J.M., Arachea, B.T., Epling, L.B., and Enemark, E.J. (2014). Analysis of the crystal structure of an active MCM hexamer. eLife *3*, e03433.

Moreno, S.P., Bailey, R., Campion, N., Herron, S., and Gambus, A. (2014). Polyubiquitylation drives replisome disassembly at the termination of DNA replication. Science *346*, 477–481.

Morohashi, H., Maculins, T., and Labib, K. (2009). The amino-terminal TPR domain of Dia2 tethers SCF(Dia2) to the replisome progression complex. Curr. Biol. *19*, 1943–1949.

Mosbech, A., Gibbs-Seymour, I., Kagias, K., Thorslund, T., Beli, P., Povlsen, L., Nielsen, S.V., Smedegaard, S., Sedgwick, G., Lukas, C., et al. (2012). DVC1 (C1orf124) is a DNA damage-targeting p97 adaptor that promotes ubiquitin-dependent responses to replication blocks. Nat. Struct. Mol. Biol. *19*, 1084–1092.

Moyer, S.E., Lewis, P.W., and Botchan, M.R. (2006). Isolation of the Cdc45/Mcm2-7/GINS (CMG) complex, a candidate for the eukaryotic DNA replication fork helicase. Proc. Natl. Acad. Sci. USA *103*, 10236–10241.

Nie, M., Aslanian, A., Prudden, J., Heideker, J., Vashisht, A.A., Wohlschlegel, J.A., Yates, J.R., 3rd, and Boddy, M.N. (2012). Dual recruitment of Cdc48 (p97)-Ufd1-Npl4 ubiquitin-selective segregase by small ubiquitin-like modifier protein (SUMO) and ubiquitin in SUMO-targeted ubiquitin ligase-mediated genome stability functions. J. Biol. Chem. *287*, 29610–29619.

Nishimura, K., Fukagawa, T., Takisawa, H., Kakimoto, T., and Kanemaki, M. (2009). An auxin-based degron system for the rapid depletion of proteins in nonplant cells. Nat. Methods *6*, 917–922.

Pacek, M., Tutter, A.V., Kubota, Y., Takisawa, H., and Walter, J.C. (2006). Localization of MCM2-7, Cdc45, and GINS to the site of DNA unwinding during eukaryotic DNA replication. Mol. Cell *21*, 581–587.

Park, S., Isaacson, R., Kim, H.T., Silver, P.A., and Wagner, G. (2005). Ufd1 exhibits the AAA-ATPase fold with two distinct ubiquitin interaction sites. Structure *13*, 995–1005.

Raman, M., Havens, C.G., Walter, J.C., and Harper, J.W. (2011). A genome-wide screen identifies p97 as an essential regulator of DNA damage-dependent CDT1 destruction. Mol. Cell *44*, 72–84.

Rappsilber, J., Ishihama, Y., and Mann, M. (2003). Stop and go extraction tips for matrix-assisted laser desorption/ionization, nanoelectrospray, and LC/MS sample pretreatment in proteomics. Anal. Chem. *75*, 663–670.

Semlow, D.R., Zhang, J., Budzowska, M., Drohat, A.C., and Walter, J.C. (2016). Replication-dependent unhooking of DNA interstrand cross-links by the NEIL3 glycosylase. Cell *167*, 498–511.

Shevchenko, A., Tomas, H., Havlis, J., Olsen, J.V., and Mann, M. (2006). In-gel digestion for mass spectrometric characterization of proteins and proteomes. Nat. Protoc. *1*, 2856–2860.

Sonneville, R., Priego-Moreno, S., Knebel, A., Johnson, C., Hastie, J., Gartner, A., Gambus, A., and Labib, K. (2017). CUL-2$^{LRR-1}$ and UBXN-3 drive replisome disassembly during DNA replication termination and mitosis. Nat. Cell Biol. Published online April 3 2017. http://dx.doi.org/10.1038/ncb3500.

Tanaka, S., Miyazawa-Onami, M., Iida, T., and Araki, H. (2015). iAID: an improved auxin-inducible degron system for the construction of a 'tight' conditional mutant in the budding yeast Saccharomyces cerevisiae. Yeast *32*, 567–581.

Vaz, B., Halder, S., and Ramadan, K. (2013). Role of p97/VCP (Cdc48) in genome stability. Front. Genet. *4*, 60.

Verma, R., Oania, R., Fang, R., Smith, G.T., and Deshaies, R.J. (2011). Cdc48/p97 mediates UV-dependent turnover of RNA Pol II. Mol. Cell *41*, 82–92.

Wang, B., Alam, S.L., Meyer, H.H., Payne, M., Stemmler, T.L., Davis, D.R., and Sundquist, W.I. (2003). Structure and ubiquitin interactions of the conserved zinc finger domain of Npl4. J. Biol. Chem. *278*, 20225–20234.

**Cell Reports, Volume *18***

## Supplemental Information

# Ufd1-Npl4 Recruit Cdc48 for Disassembly of Ubiquitylated CMG Helicase at the End of Chromosome Replication

**Marija Maric, Progya Mukherjee, Michael H. Tatham, Ronald Hay, and Karim Labib**

Maric et al Supplementary Figure 1, related to Figure 1

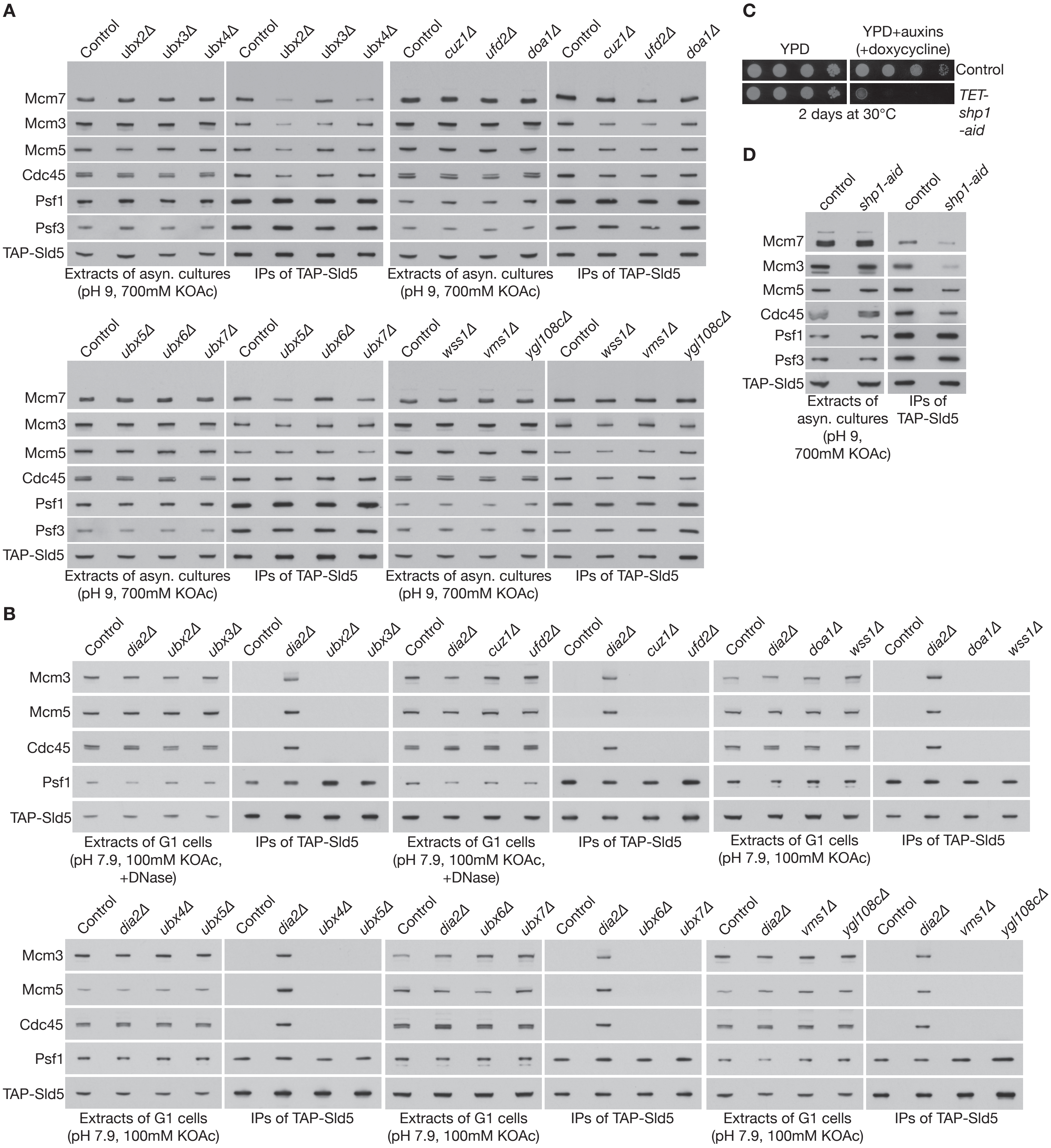


Screen for adaptors of Cdc48 that are required for disassembly of ubiquitylated CMG helicase at the end of chromosome replication. (**A**) Asynchronous cultures of the indicated strains were grown at 30°C. The presence of ubiquitylated CMG helicase was monitored by immunoprecipitation of TAP-Sld5 from high salt extracts, as in Figure 1 for *ufd1-2* and *npl4-1* (which provide a positive control for the factors shown here). (**B**) The indicated strains were grown at 30°C and then arrested in G1-phase. The persistence of old CMG complexes was monitored by immunoprecipitation of TAP-Sld5 from cell extracts - *dia2Δ* provided a positive control for defective CMG disassembly. (**C**) Comparison of growth of control cells (YMM573) and TET-shp1-aid (YMM591) on rich medium (YPD) in the presence or absence of auxins (to induce degradation of Shp1-aid) and doxycycline (to repress expression of *shp1-aid* from the *TET* promoter). (**D**) Cells were grown at 30°C before addition of doxycycline and auxin for 3 hours (control strain YSS47 and *TET-shp1-aid* strain YMM591). The presence of ubiquitylated CMG helicase was assayed as above.

Maric et al Supplementary Figure 2, related to Figure 1

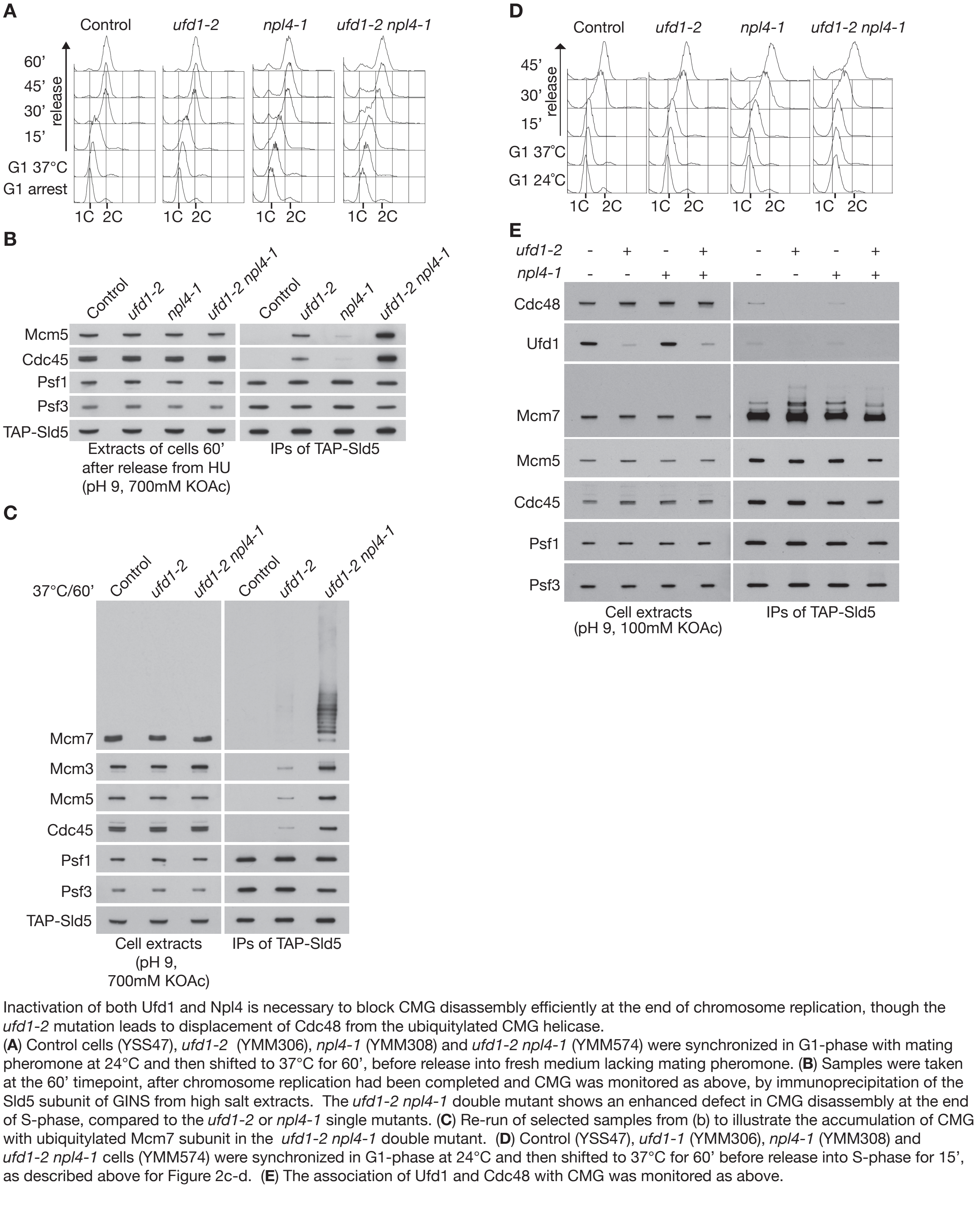


Inactivation of both Ufd1 and Npl4 is necessary to block CMG disassembly efficiently at the end of chromosome replication, though the *ufd1-2* mutation leads to displacement of Cdc48 from the ubiquitylated CMG helicase.
(**A**) Control cells (YSS47), *ufd1-2* (YMM306), *npl4-1* (YMM308) and *ufd1-2 npl4-1* (YMM574) were synchronized in G1-phase with mating pheromone at 24°C and then shifted to 37°C for 60', before release into fresh medium lacking mating pheromone. (**B**) Samples were taken at the 60' timepoint, after chromosome replication had been completed and CMG was monitored as above, by immunoprecipitation of the Sld5 subunit of GINS from high salt extracts. The *ufd1-2 npl4-1* double mutant shows an enhanced defect in CMG disassembly at the end of S-phase, compared to the *ufd1-2* or *npl4-1* single mutants. (**C**) Re-run of selected samples from (b) to illustrate the accumulation of CMG with ubiquitylated Mcm7 subunit in the *ufd1-2 npl4-1* double mutant. (**D**) Control (YSS47), *ufd1-1* (YMM306), *npl4-1* (YMM308) and *ufd1-2 npl4-1* cells (YMM574) were synchronized in G1-phase at 24°C and then shifted to 37°C for 60' before release into S-phase for 15', as described above for Figure 2c-d. (**E**) The association of Ufd1 and Cdc48 with CMG was monitored as above.

Maric et al Supplementary Figure 3, related to Figure 3

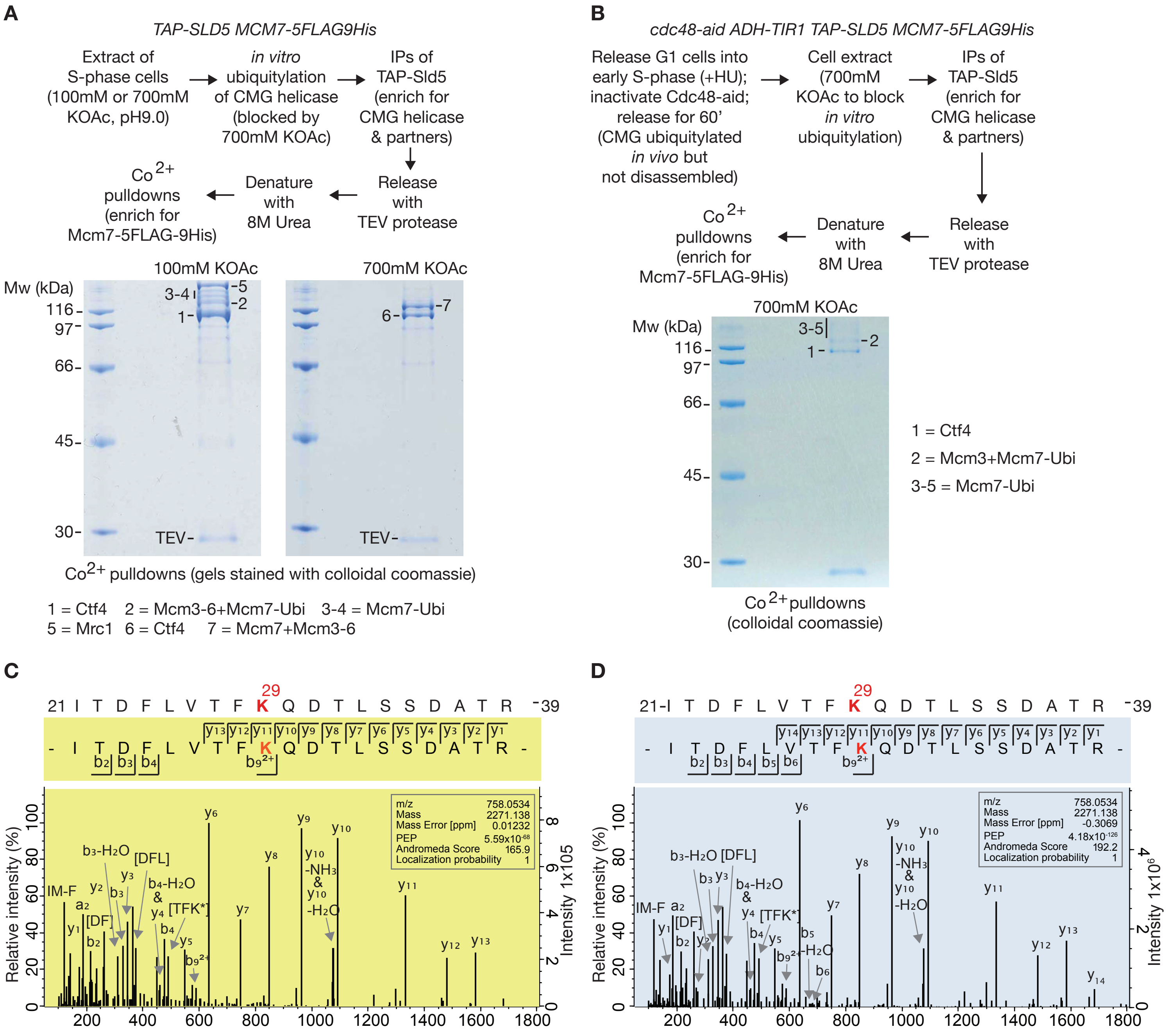


Lysine 29 of Mcm7 is the major site of CMG ubiquitylation both *in vitro* and *in vivo*.
(**A**) Scheme to enrich for ubiquitylated Mcm7 from isolated CMG helicase complexes. After isolation of GINS (and thus CMG) by immunoprecipitation of TAP-Sld5 from extracts of S-phase cells (YGDP483), we released the bound complexes with TEV protease, denatured the material with 8M urea, and then isolated Mcm7-5FLAG9His on cobalt-coated magnetic beads. We compared low salt extracts that supported *in vitro* ubiquitylation with high salt extracts in which ubiquitylation was blocked. Note that several components of CMG, or associated factors, bound non-specifically in their denatured form to the cobalt-coated beads (Ctf4, Mrc1, Mcm3, Mcm6 - the presence of the latter factors was greatly reduced in high salt extracts). The purified material was then resolved in a 10% gel, and the indicated bands excised for mass spectrometry analysis. (**B**) Similar analysis of Mcm7 from in vivo ubiquitylated CMG helicase. A culture of *cdc48-aid* cells (YMM228) was synchronised in G1-phase with mating pheromone and then arrested in early S-phase with 0.2M hydroxyurea, before depletion of Cdc48-aid by addition of auxin for 60'. Cells were then washed into fresh medium lacking hydroxyurea, and incubated for a further 60'. (**C**) Following in-gel digestion of in vitro ubiquitylated Mcm7 with trypsin and GluC, mass spectrometry analysis indicated that K29 was the only detectable site of Gly-Gly modification, indicative of ubiquitylation. The panel shows the annotated MS/MS spectrum of the GluC/Trypsin peptide that is diagnostic of ubiquitylated Mcm7-K29 (only the peaks with >10% of maximum intensity are annotated; annotations in square brackets show internal peptide fragments). (**D**) *In vivo* ubiquitylated Mcm7 was processed in the same way, and once again K29 was the only detectable site of ubiquitylation.

# Maric et al Supplementary Figure 4, related to Figure 4

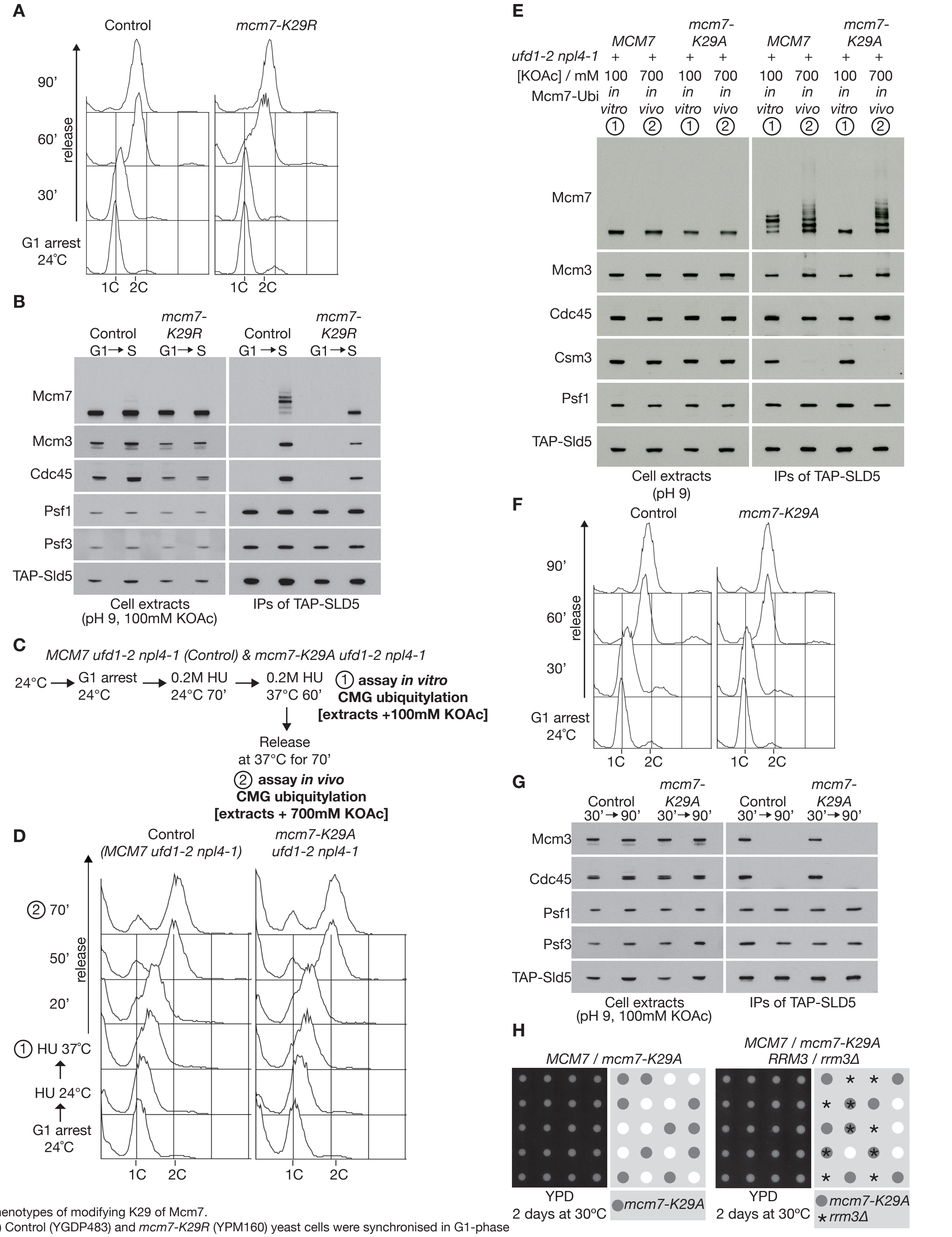


Phenotypes of modifying K29 of Mcm7.

(**A**) Control (YGDP483) and *mcm7-K29R* (YPM160) yeast cells were synchronised in G1-phase at 24°C andthen released into S-phase for 20’. (**B**) *In vitro* ubiquitylation of the CMG helicase in extracts of S-phase cells was monitored as above.

(**C**) *MCM7 ufd1-2 npl4-1* control cells (YPM81) and *mcm7-K29a ufd1-2 npl4-1* (YPM94) were grown as in Figure 1c. Cells were synchronized in G1-phase at 24°C and then released into S-phase in the presence of 0.2M hydroxyurea for 70’, to allow for CMG assembly at forks from early origins of replication. The cells were then shifted to 37°C for 60’ to inactivate Ufd1-Npl4, before washing into fresh medium lacking hydroxyurea, to allow cells to complete chromosome replication. Sample 1 corresponded to HU arrested cells at 37°C that lacked in vivo CMG ubiquitylation (defective progression of DNA replication forks blocked the termination of DNA replication), and was used to prepare ‘low-salt extracts’ in order to monitor in vitro ubiquitylation of CMG helicase. In contrast, Sample 2 was taken at the end of the experiment and corresponded to cells that had completed chromosome replication in the absence of CMG disassembly (due to inactivation of Ufd1-Npl4); this sample was used to prepare ‘high-salt’ extracts in order to monitor in vivo ubiquitylated CMG helicase. (**D**) DNA content was monitored throughout the experiment by flow cytometry. (**E**) The CMG helicase was isolated from cell extracts by immunoprecipitation of the Sld5 subunit of GINS, revealing that the mcm7-K29A mutation abolished in vitro CMG ubiquitylation in cell extracts but did not prevent in vivo ubiquitylation at the end of chromosome replication. (**F**) Synchronised cultures of control (YPM81) and *mcm7-K29A* cells (YPM94) were generated as in Supplementary Fig. 6. (**G**) CMG disassembly was monitored by immunoprecipitation of the Sld5 subunit of GINS in mid-S-phase (30’ release from G1-phase) and following the completion of chromosome replication (90’). (**H**) Tetrad analysis of the indicated diploid strains (YMM476 and YMM482) shows that *mcm7-K29A* grows like control cells and is not synthetic lethal with *rrm3Δ*.

Maric et al Supplementary Table 1, related to Figure 3

| | *In vitro* Mcm7-Ubi | | *In vivo* Mcm7-Ubi | |
|---|---|---|---|---|
| **Fragment** | **Mass Deviation (ppm)** | **Mass (Da)** | **Mass Deviation (ppm)** | **Mass (Da)** |
| $y_1$ | 1.6420 | 175.1187 | 3.9000 | 175.1183 |
| $a_2$ | 1.6948 | 187.1438 | 3.8979 | 187.1434 |
| $b_2$-$H_2O$ | 1.1290 | 197.1282 | 3.5890 | 197.1277 |
| $b_2$ | 1.7325 | 215.1386 | 3.8394 | 215.1382 |
| $y_2$-$NH_3$ | | | 4.2236 | 259.1390 |
| $y_2$ | 0.8279 | 276.1664 | 1.6296 | 276.1662 |
| $b_3$-$H_2O$ | 3.0079 | 312.1545 | 5.2404 | 312.1538 |
| $b_3$ | 4.6527 | 330.1644 | 7.3189 | 330.1635 |
| $y_3$ | 1.8004 | 347.2031 | 3.8534 | 347.2024 |
| $b_4$-$H_2O$ | 1.9465 | 459.2229 | | |
| $y_4$ | 1.9004 | 462.2298 | 3.9021 | 462.2289 |
| $b_4$ | 1.5331 | 477.2336 | 4.0143 | 477.2325 |
| $y_5$ | 0.6584 | 549.2624 | 3.6980 | 549.2607 |
| $b_5$-$H_2O$ | | | 0.8942 | 572.3074 |
| $b_9^{(2+)}$ | 0.1526 | 590.3240 | 2.8573 | 590.3224 |
| $y_6$ | 1.8132 | 636.2936 | 3.9031 | 636.2923 |
| $b_6$ | | | 1.6684 | 689.3857 |
| $y_7$ | 2.8235 | 749.3767 | 4.2774 | 749.3756 |
| $y_8$ | 2.2385 | 850.4246 | 3.6823 | 850.4234 |
| $y_9$ | 2.2967 | 965.4512 | 4.0344 | 965.4495 |
| $y_{10}$-$H_2O$ | 1.9836 | 1075.4993 | 3.6085 | 1075.4976 |
| $y_{10}$-$NH_3$ | -1.4574 | 1076.4870 | 0.7890 | 1076.4846 |
| $y_{10}$ | 1.8315 | 1093.5100 | 3.7835 | 1093.5079 |
| $y_{11}$ | 1.3399 | 1335.6481 | 3.1013 | 1335.6458 |
| $y_{12}$-$H_2O$ | | | 0.9205 | 1464.7064 |
| $y_{12}$-$NH_3$ | | | 2.7067 | 1465.6878 |
| $y_{12}$ | 1.4668 | 1482.7161 | 3.3160 | 1482.7134 |
| $y_{13}$-$H_2O$ | | | 3.1593 | 1565.7505 |
| $y_{13}$-$NH_3$ | | | -0.5000 | 1566.7402 |
| $y_{13}$ | 0.6992 | 1583.7649 | 3.2874 | 1583.7608 |
| $y_{14}$ | | | 1.1983 | 1682.8324 |

Summary of MS data for mapping of Mcm7 ubiquitylation sites.
The table shows the annotated MS/MS data for the two spectra in Figure S3C-D.

| Strain | Genotype |
|---|---|
| W303-1 | *MAT***a** *ade2-1 ura3-1 his3-11,15 trp1-1 leu2-3,112 can1-100 / MAT*$\alpha$ *ade2-1 ura3-1 his3-11,15 trp1-1 leu2-3,112 can1-100* |
| YASD375 | *MAT***a** *TAP-SLD5 (kanMX) pep4Δ::URA3 ADE2* |
| YGDP483 | *MAT***a** *TAP-SLD5 (kanMX) MCM7-5FLAG9His (hphNT) pep4Δ::URA3 ADE2* |
| YMM120 | *MAT***a** *leu2::pRS305-GAL-MCM7 mcm7-td (K.l.TRP1) GAL-UBR1 (URA3) TAP-SLD5 (kanMX) pep4Δ::ADE2* |
| YMM228 | *MAT***a** *cdc48-aid (hphNT) ADH1-OsTIR1 (URA3 & K.l.TRP1) TAP-SLD5 (kanMX) pep4Δ::URA3 ADE2* |
| YMM233 | *MAT***a** *leu2::pRS305-GAL-mcm7-K624A,K637A,K648A,K672A,K678A mcm7-td (K.l.TRP1) GAL-UBR1 (URA3) TAP-SLD5 (kanMX) pep4Δ::ADE2* |
| YMM238 | *MAT***a** *leu2::pRS305-GAL-mcm7-K442A,K449R,K471A,K475R,K486R,K499R,K528A,K550A mcm7-td (K.l.TRP1) GAL-UBR1 (URA3) TAP-SLD5 (kanMX) pep4Δ::ADE2* |
| YMM240 | *MAT***a** *leu2::pRS305-GAL-mcm7-K733A,K735A,K743A,K749A,K750A,K757A,K767A,K822A mcm7-td (K.l.TRP1) GAL-UBR1 (URA3) TAP-SLD5 (kanMX) pep4Δ::ADE2* |
| YMM242 | *MAT***a** *leu2::pRS305-GAL-mcm7-K29A,K59A,K62A,K69A,K92A,K130A,K135A mcm7-td (K.l.TRP1) GAL-UBR1 (URA3) TAP-SLD5 (kanMX) pep4Δ::ADE2* |
| YMM244 | *MAT***a** *leu2::pRS305-GAL-mcm7-K217A,K218A,K223A,K231A,K252R,K295A,K314R,K364A,K367A,K385A,K386R,K387A mcm7-td (K.l.TRP1) GAL-UBR1 (URA3) TAP-SLD5 (kanMX) pep4Δ::ADE2* |
| YMM306 | *MAT***a** *ufd1-2 TAP-SLD5 (kanMX) pep4Δ::URA3 ADE2* |
| YMM308 | *MAT***a** *npl4-1 TAP-SLD5 (kanMX) pep4Δ::URA3 ADE2* |
| YMM377 | *MAT***a** *leu2::pRS305-GAL-mcm7-K29A,K59A,K62A mcm7-td (K.l.TRP1) GAL-UBR1 (URA3) TAP-SLD5 (kanMX) pep4Δ::ADE2* |
| YMM379 | *MAT***a** *leu2::pRS305-GAL-mcm7-K29A,K59A,K62A,K69A mcm7-td (K.l.TRP1) GAL-UBR1 (URA3) TAP-SLD5 (kanMX) pep4Δ::ADE2* |
| YMM396 | *MAT***a** *leu2::pRS305-GAL-mcm7-K29A,K59A,K62A,K69A,K92A mcm7-td (K.l.TRP1) GAL-UBR1 (URA3) TAP-SLD5 (kanMX) pep4Δ::ADE2* |
| YMM397 | *MAT***a** *leu2::pRS305-GAL-mcm7-K130A,K135A mcm7-td (K.l.TRP1) GAL-UBR1 (URA3) TAP-SLD5 (kanMX) pep4Δ::ADE2* |
| YMM408 | *MAT***a** *ubx5Δ::K.l.TRP1 TAP-SLD5 (kanMX) pep4Δ::ADE2* |
| YMM412 | *MAT***a** *ufd2Δ::K.l.TRP1 TAP-SLD5 (kanMX) pep4Δ::ADE2* |
| YMM435 | *MAT***a** *leu2::pRS305-GAL-mcm7-K29A mcm7-td (K.l.TRP1) GAL-UBR1 (URA3) TAP-SLD5 (kanMX) pep4Δ::ADE2* |
| YMM437 | *MAT***a** *leu2::pRS305-GAL-mcm7-K59A mcm7-td (K.l.TRP1) GAL-UBR1 (URA3) TAP-SLD5 (kanMX) pep4Δ::ADE2* |
| YMM439 | *MAT***a** *leu2::pRS305-GAL-mcm7-K62A mcm7-td (K.l.TRP1) GAL-* |

| | |
|---|---|
| | *UBR1 (URA3) TAP-SLD5 (kanMX) pep4Δ::ADE2* |
| YMM458 | *MAT***a** *cuz1Δ::K.l.TRP1 TAP-SLD5 (kanMX) pep4Δ::ADE2* |
| YMM460 | *MAT***a** *ubx4Δ::K.l.TRP1 TAP-SLD5 (kanMX) pep4Δ::ADE2* |
| YMM476 | *MAT***a** / *MATα MCM7 / mcm7-K29A-5FLAG9His (hphNT)* |
| YMM482 | *MAT***a** / *MATα MCM7 / mcm7-K29A-5FLAG9His (hphNT) RRM3 / rrm3Δ* |
| YMM493 | *MAT***a** *mcm7-K29A-5FLAG9His (hphNT) TAP-SLD5 (kanMX) pep4Δ::ADE2* |
| YMM495 | *MAT***a** *vms1Δ::K.l.TRP1 TAP-SLD5 (kanMX) pep4Δ::ADE2* |
| YMM503 | *MAT***a** *ygl108cΔ::K.l.TRP1 TAP-SLD5 (kanMX) pep4Δ::ADE2* |
| YMM510 | *MAT***a** / *MATα UBX5 / ubx5Δ::K.l.TRP1 DIA2 / dia2Δ::HIS3MX* |
| YMM511 | *MAT***a** / *MATα UFD2 / ufd2Δ::K.l.TRP1 DIA2 / dia2Δ::HIS3MX* |
| YMM512 | *MAT***a** / *MATα CUZ1 / cuz1Δ::K.l.TRP1 DIA2 / dia2Δ::HIS3MX* |
| YMM513 | *MAT***a** / *MATα YGL108C / ygl108cΔ::K.l.TRP1 DIA2 / dia2Δ::HIS3MX* |
| YMM530 | *MAT***a** / *MATα UBX2 / ubx2Δ::hphNT DIA2 / dia2Δ::HIS3MX* |
| YMM531 | *MAT***a** / *MATα UBX3 / ubx3Δ::HIS3MX DIA2 / dia2Δ::HIS3MX* |
| YMM532 | *MAT***a** / *MATα UBX6 / ubx6Δ::URA3CP DIA2 / dia2Δ::HIS3MX* |
| YMM533 | *MAT***a** / *MATα UBX7 / ubx7Δ::HIS3MX DIA2 / dia2Δ::HIS3MX* |
| YMM534 | *MAT***a** / *MATα UBX4 / ubx4Δ::K.l.TRP1 DIA2 / dia2Δ::HIS3MX* |
| YMM543 | *MAT***a** *ubx2Δ::hphNT TAP-SLD5 (kanMX) pep4Δ::ADE2* |
| YMM545 | *MAT***a** *ubx3Δ::HIS3MX TAP-SLD5 (kanMX) pep4Δ::ADE2* |
| YMM547 | *MAT***a** *ubx6Δ::URA3CP TAP-SLD5 (kanMX) pep4Δ::ADE2* |
| YMM549 | *MAT***a** *ubx7Δ::HIS3MX TAP-SLD5 (kanMX) pep4Δ::ADE2* |
| YMM555 | *MAT***a** / *MATα DOA1 / doa1Δ::hphNT DIA2 / dia2Δ::HIS3MX* |
| YMM556 | *MAT***a** / *MATα WSS1 / wss1Δ::hphNT DIA2 / dia2Δ::HIS3MX* |
| YMM557 | *MAT***a** *doa1Δ::hphNT TAP-SLD5 (kanMX) pep4Δ::ADE2* |
| YMM559 | *MAT***a** *wss1Δ::hphNT TAP-SLD5 (kanMX) pep4Δ::ADE2* |
| YMM561 | *MAT***a** / *MATα VMS1 / vms1Δ::K.l.TRP1 DIA2 / dia2Δ::HIS3MX* |
| YMM562 | *MAT***a** / *MATα UFD1 / ufd1-2 DIA2 / dia2Δ::HIS3MX* |
| YMM563 | *MAT***a** / *MATα NPL4 / npl4-1 DIA2 / dia2Δ::HIS3MX* |
| YMM573 | *MATα leu2::pCM244 ADH1-OsTIR1 (URA3 & K.l.TRP1) TAP-SLD5 pep4Δ::ADE2* |
| YMM574 | *MAT***a** *ufd1-2 npl4-1 MCM7-5FLAG9His (hphNT) TAP-SLD5 (kanMX) pep4Δ::URA3 ADE2* |
| YMM591 | *MAT***a** *TET-shp1-aid (kanMX & hphNT) leu2::pCM244 ADH1-OsTIR1 (URA3 & K.l.TRP1) TAP-SLD5 (kanMX) pep4Δ::ADE2* |
| YPM81 | *MAT***a** *ufd1-2 npl4-1 TAP-SLD5 (kanMX) pep4Δ::ADE2* |
| YPM94 | *MAT***a** *ufd1-2 npl4-1 mcm7-K29A-5FLAG9His (hphNT) TAP-SLD5 (kanMX) pep4Δ::ADE2* |
| YPM160 | *MAT***a** *mcm7-K29R-5FLAG9His (hphNT) TAP-SLD5 (kanMX) pep4Δ::ADE2* |
| YSS47 | *MAT***a** *TAP-SLD5 (kanMX) pep4Δ::ADE2* |
| YSS125 | *MAT***a** *mcm7-td (K.l.TRP1) GAL-UBR1 (URA3) TAP-SLD5 (kanMX) pep4Δ::ADE2* |
| YTM572 | *MAT***a** *dia2Δ::HIS3MX TAP-SLD5 (kanMX) pep4Δ::ADE2* |

**Supplementary Table 2, related to Figures 1-4**

Yeast strains used in this study.  All strains are based on the W303 background.
